# Supplementary material for: Preclinical evaluation of CRISPR-based therapies for Noonan syndrome caused by deep-intronic LZTR1 variants
Source: Mol Ther Nucleic Acids. 2024 Jan 23;35(1):102123. doi: 10.1016/j.omtn.2024.102123 (PMC10851011; doi:10.1016/j.omtn.2024.102123)
Supplement: Document S1. Figures S1–S7 and Tables S1–S4 [file mmc1.pdf]

OMTN, Volume *35*

Supplemental information

Preclinical evaluation of CRISPR-based therapies for Noonan syndrome caused by deep-intronic *LZTR1* variants

Carolin Knauer, Henrike Haltern, Eric Schoger, Sebastian Kügler, Lennart Roos, Laura C. Zelarayán, Gerd Hasenfuss, Wolfram-Hubertus Zimmermann, Bernd Wollnik, and Lukas Cyganek

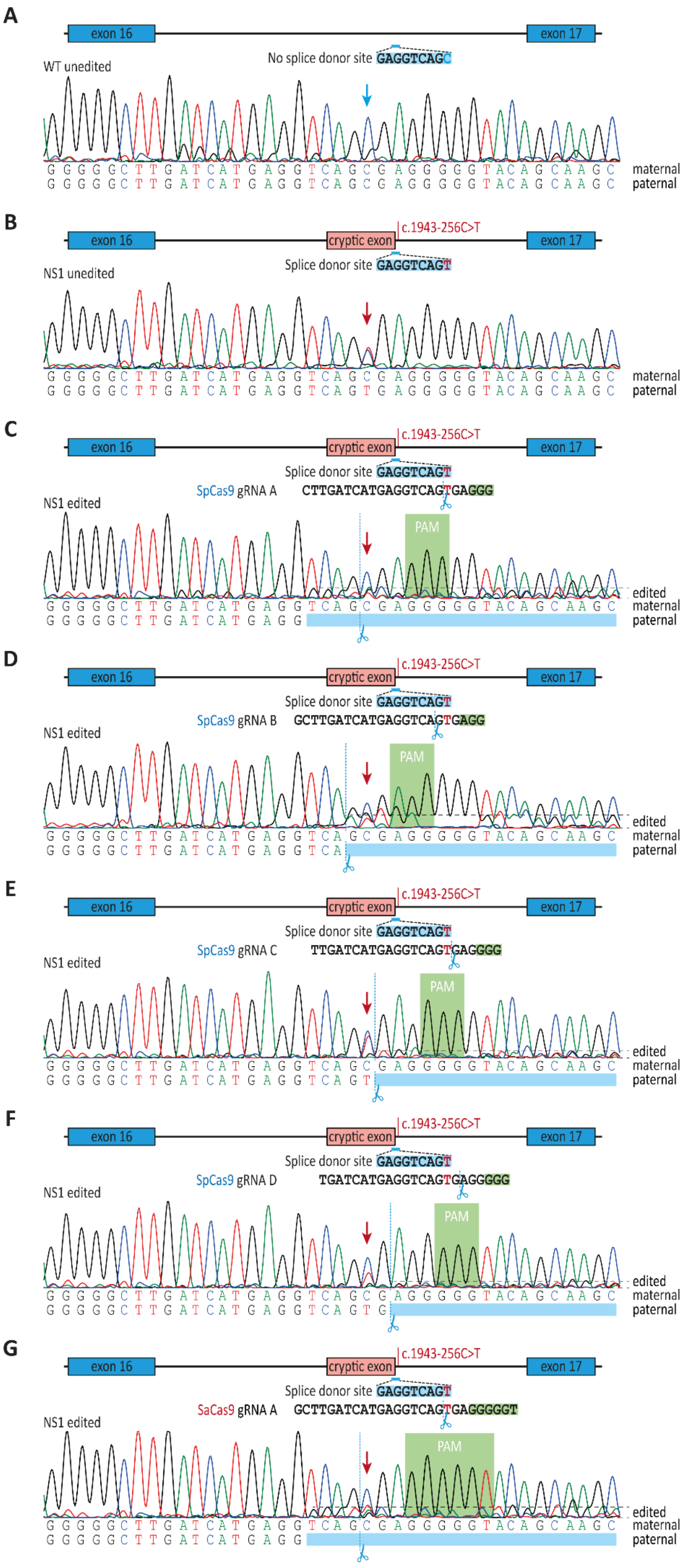

A
exon 16
exon 17
No splice donor site GAGGTCAGC
WT unedited
GGGGGCTTGATCATGAGGTCAGCGAGGGGGTACAGCAAGC
GGGGGCTTGATCATGAGGTCAGCGAGGGGGTACAGCAAGC
maternal
paternal
B
exon 16
cryptic exon
c.1943-256C>T
exon 17
Splice donor site GAGGTCAGT
NS1 unedited
GGGGGCTTGATCATGAGGTCAGCGAGGGGGTACAGCAAGC
GGGGGCTTGATCATGAGGTCAGTGAGGGGGTACAGCAAGC
maternal
paternal
C
exon 16
cryptic exon
c.1943-256C>T
exon 17
Splice donor site GAGGTCAGT
SpCas9 gRNA A CTTGATCATGAGGTCAGTGAGGG
NS1 edited
PAM
GGGGGCTTGATCATGAGGTCAGCGAGGGGGTACAGCAAGC
GGGGGCTTGATCATGAGG
edited
maternal
paternal
D
exon 16
cryptic exon
c.1943-256C>T
exon 17
Splice donor site GAGGTCAGT
SpCas9 gRNA B GCTTGATCATGAGGTCAGTGAGG
NS1 edited
PAM
GGGGGCTTGATCATGAGGTCAGCGAGGGGGTACAGCAAGC
GGGGGCTTGATCATGAGGTCA
edited
maternal
paternal
E
exon 16
cryptic exon
c.1943-256C>T
exon 17
Splice donor site GAGGTCAGT
SpCas9 gRNA C TTGATCATGAGGTCAGTGAGGGG
NS1 edited
PAM
GGGGGCTTGATCATGAGGTCAGCGAGGGGGTACAGCAAGC
GGGGGCTTGATCATGAGGTCAGT
edited
maternal
paternal
F
exon 16
cryptic exon
c.1943-256C>T
exon 17
Splice donor site GAGGTCAGT
SpCas9 gRNA D TGATCATGAGGTCAGTGAGGGGG
NS1 edited
PAM
GGGGGCTTGATCATGAGGTCAGCGAGGGGGTACAGCAAGC
GGGGGCTTGATCATGAGGTCAGTG
edited
maternal
paternal
G
exon 16
cryptic exon
c.1943-256C>T
exon 17
Splice donor site GAGGTCAGT
SaCas9 gRNA A GCTTGATCATGAGGTCAGTGAGGGGGT
NS1 edited
PAM
GGGGGCTTGATCATGAGGTCAGCGAGGGGGTACAGCAAGC
GGGGGCTTGATCATGAGG
edited
maternal
paternal

Figure S1: Allele-specific targeting of the deep-intronic variant in *LZTR1* intron 16 by different CRISPR/Cas9 combinations in patient-specific iPSCs. (A,B) Sanger sequencing of *LZTR1* intron 16 for WT (A) and NS1 (B). **(C-G)** Analysis of editing efficiencies of iPSCs from patient NS1 3-4 days post-transfection by Sanger sequencing for allele-specific disruption of the cryptic splice site using SpCas9 with guide RNA A (C), SpCas9 with guide RNA B (D), SpCas9 with guide RNA C (E), SpCas9 with guide RNA D (F), and SaCas9 with guide RNA A (G).

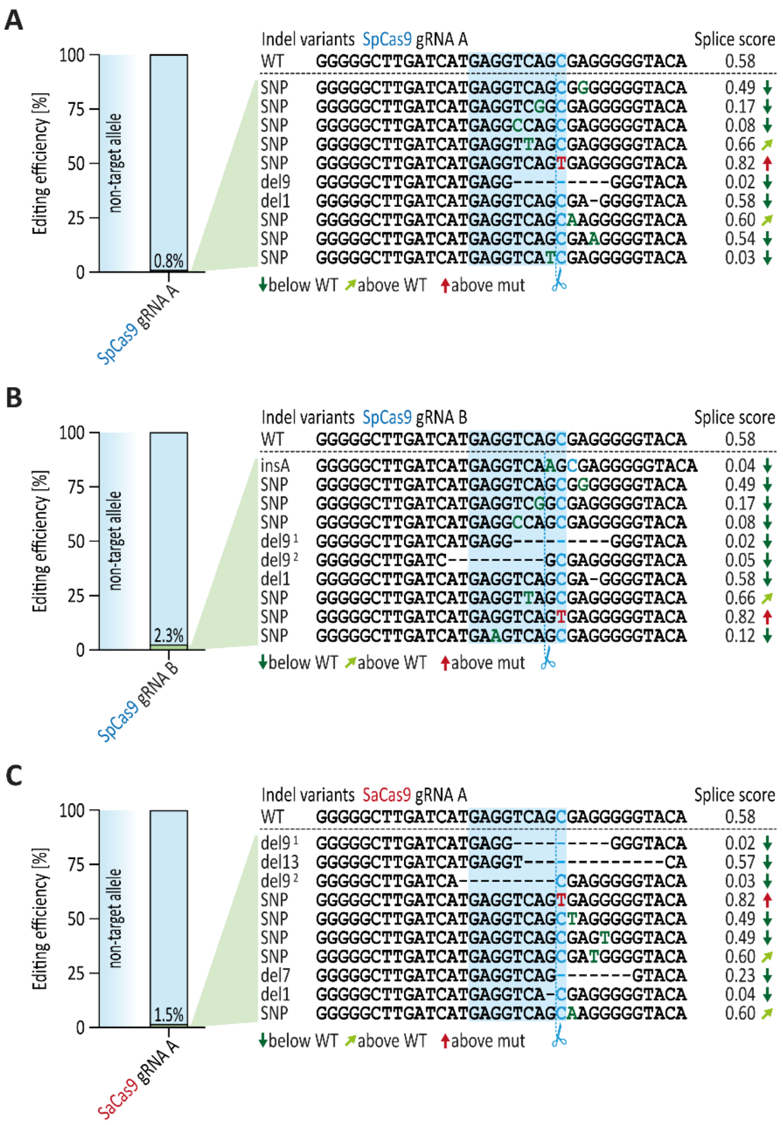

Figure S2: Evaluation of editing specificity of the different CRISPR/Cas9 combinations targeting *LZTR1* intron 16 in WT iPSCs. (A-C) Analysis of off-target editing of the non-mutated *LZTR1* intron 16 locus in WT iPSCs 3-4 days post-transfection using amplicon sequencing and computational prediction of splice site motifs for SpCas9 and guide RNA A (A), for SpCas9 and guide RNA B (B), and for SaCas9 and guide RNA A (C).

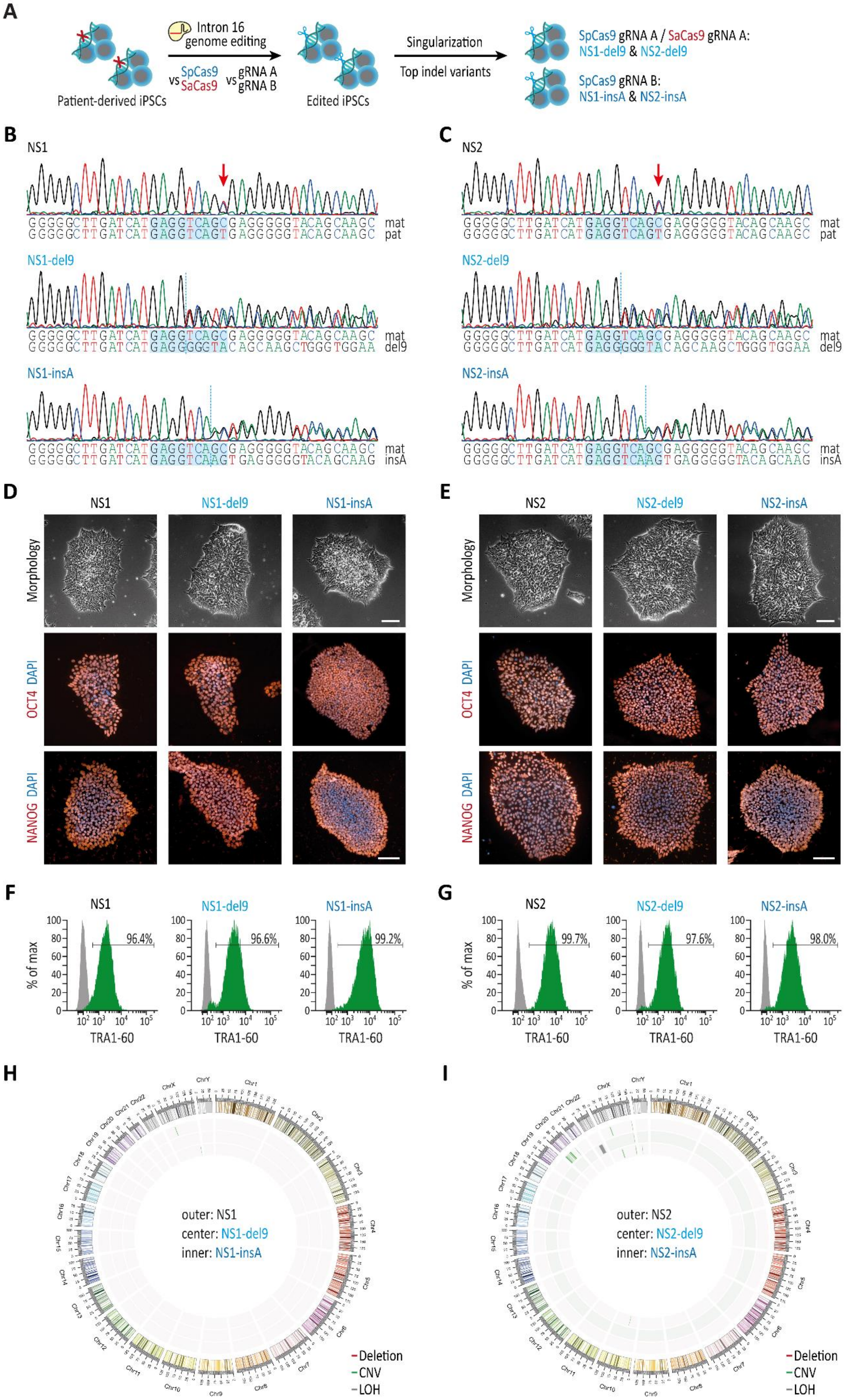
A
Patient-derived iPSCs
Intron 16 genome editing
vs SpCas9 SaCas9
vs gRNA A gRNA B
Edited iPSCs
Singularization
Top indel variants
SpCas9 gRNA A / SaCas9 gRNA A: NS1-del9 & NS2-del9
SpCas9 gRNA B: NS1-insA & NS2-insA
B
NS1
GGGGGCTTGATCATGAGGTCAGCGAGGGGGTACAGCAAGC mat
GGGGGCTTGATCATGAGGTCAGTGAGGGGGTACAGCAAGC pat
NS1-del9
GGGGGCTTGATCATGAGGTCAGCGAGGGGGTACAGCAAGC mat
GGGGGCTTGATCATGAGGGGGTACAGCAAGCTGGGTGGAA del9
NS1-insA
GGGGGCTTGATCATGAGGTCAGCGAGGGGGTACAGCAAGC mat
GGGGGCTTGATCATGAGGTCAAGTGAGGGGGTACAGCAAG insA
C
NS2
GGGGGCTTGATCATGAGGTCAGCGAGGGGGTACAGCAAGC mat
GGGGGCTTGATCATGAGGTCAGTGAGGGGGTACAGCAAGC pat
NS2-del9
GGGGGCTTGATCATGAGGTCAGCGAGGGGGTACAGCAAGC mat
GGGGGCTTGATCATGAGGGGGTACAGCAAGCTGGGTGGAA del9
NS2-insA
GGGGGCTTGATCATGAGGTCAGCGAGGGGGTACAGCAAGC mat
GGGGGCTTGATCATGAGGTCAAGTGAGGGGGTACAGCAAG insA
D
NS1
NS1-del9
NS1-insA
Morphology
OCT4 DAPI
NANOG DAPI
E
NS2
NS2-del9
NS2-insA
Morphology
OCT4 DAPI
NANOG DAPI
F
NS1
96.4%
NS1-del9
96.6%
NS1-insA
99.2%
% of max
TRA1-60
G
NS2
99.7%
NS2-del9
97.6%
NS2-insA
98.0%
% of max
TRA1-60
H
outer: NS1
center: NS1-del9
inner: NS1-insA
Deletion
CNV
LOH
I
outer: NS2
center: NS2-del9
inner: NS2-insA
Deletion
CNV
LOH

Figure S3: Generation of isogenic iPSCs with top indel variants upon CRISPR/Cas9 editing of *LZTR1* intron 16 in patient-specific iPSCs. (A) Generation of monoclonal CRISPR-edited iPSCs harboring the top indel variants for SpCas9/SaCas9 with guide RNA A and SpCas9 with guide RNA B by singularization of transfected bulks. **(B,C)** Sanger sequencing of unedited and CRISPR-edited iPSCs harboring the top indel variants 9-base pair deletion (del9) and 1-base pair insertion of adenosine (insA) derived from patients NS1 (B) and NS2 (C). **(D,E)** Patient-specific and CRISPR-edited iPSCs derived from patients NS1 (D) and NS2 (E) showed a typical human stem cell-like morphology and expressed the key pluripotency markers OCT3/4 and NANOG as assessed by light microscopy and immunocytochemistry; nuclei were counter-stained with Hoechst 33342 (blue); scale bar: 100 μm. **(F,G)** Flow cytometry analysis of pluripotency marker TRA-1-60 revealed homogeneous populations of pluripotent cells in generated iPSC lines derived from patients NS1 (F) and NS2 (G). Gray peaks represent the negative controls. **(H,I)** Molecular karyotyping using a genome-wide microarray demonstrated chromosomal stability after genome editing of iPSCs derived from patients NS1 (H) and NS2 (I). CNV: copy number variation, LOH: loss of heterozygosity.

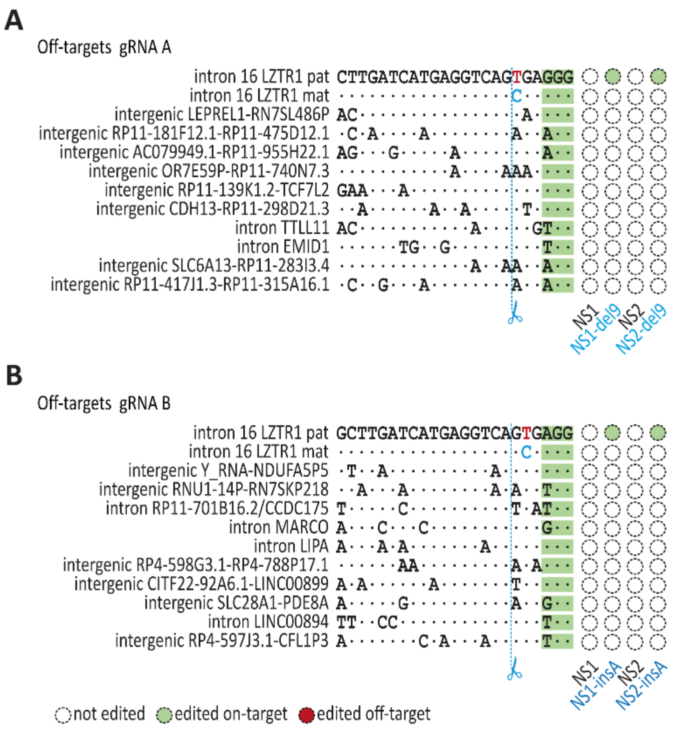

Figure S4: Off-target screening in CRISPR-edited iPSCs. (A,B) Sanger sequencing of the top ten predicted off-target regions, ranked by the CFD off-target score using CRISPOR, revealed no off-target editing of CRISPR/Cas9 in CRISPR-edited iPSCs compared to patient-derived cells for SpCas9 and guide RNA A (A) and SpCas9 and guide RNA B (B).

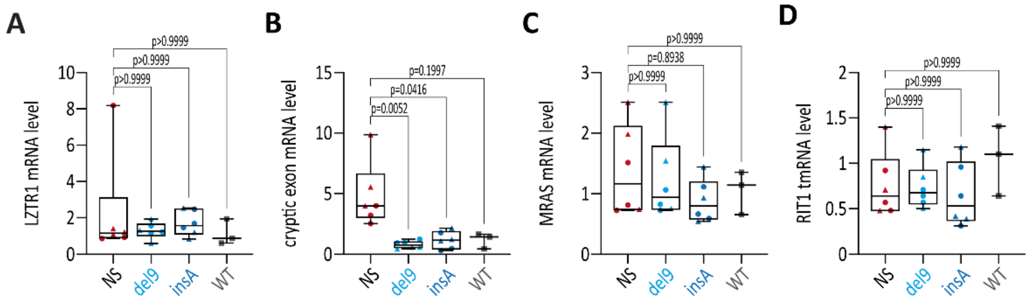

Figure S5: Gene expression analysis in patient-specific and CRISPR-edited iPSC-CMs. (A-D) Quantitative gene expression analysis of *LZTR1* (A), *LZTR1* transcript including the cryptic exon (B), and LZTR1 substrates *MRAS* (C), and *RIT1* (D) in patient-specific and CRISPR-edited iPSC-CMs at day 30 of differentiation, assessed by real-time PCR, revealed no expression differences at transcriptional level across all iPSC lines; samples were analyzed in triplicates and data were normalized to *GAPDH*, *RPL37A*, and *TUBB5* expression and WT controls; n=3 independent differentiations per iPSC line. Data were analyzed by nonparametric Kruskal-Wallis test with Dunn's correction and are presented as mean ± SEM (A-D).

A

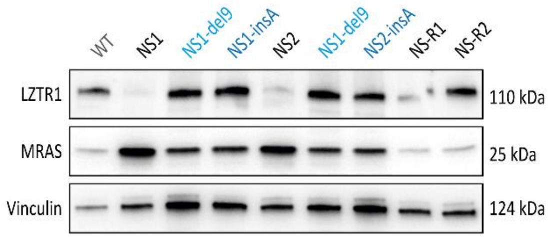

Figure S6: Restoration of LZTR1 function and normalization of MRAS levels upon CRISPR/Cas9 editing of *LZTR1* intron 16 compared to parents' iPSC-CMs. (A) Representative blots of LZTR1 and MRAS levels, assessed by Western blot, in patient-specific iPSC-CMs and CRISPR-corrected iPSC-CMs in comparison to iPSC-CMs from the father (NS-R1) and the mother (NS-R2) at day 30 of differentiation; Vinculin served as loading control.

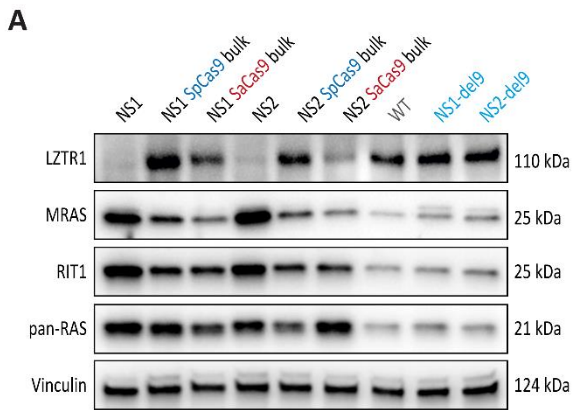

Figure S7: Restoration of LZTR1 function and normalization of RAS GTPase levels upon CRISPR/Cas9 editing of *LZTR1* intron 16 in differentiated bulks. (A) Representative blots of LZTR1 and RAS GTPase levels, assessed by Western blot, in iPSC-CMs at day 30 of differentiation transfected at iPSC level in comparison to unedited and monoclonal CRISPR-edited iPSC-CMs from the patients; Vinculin served as loading control.

Table S1: Guide RNA target sequences targeting *LZTR1* intron 16.

**Guide RNA**	**Target sequence**
guide RNA A	(G)CTTGATCATGAGGTCAGTGA
guide RNA B	GCTTGATCATGAGGTCAGTG
guide RNA C	TTGATCATGAGGTCAGTGAG
guide RNA D	TGATCATGAGGTCAGTGAGG

Table S2: Primer sequences used for PCR, reverse transcriptase PCR and real-time PCR.

**Gene (gDNA)**	**Primer**
LZTR1 In16	TGTAATGTCACAGGCCCCTT /<br>TGTTGCTGCCCTGAACAAAA
Guide RNA A<br>Off-target 1	GCATAAAAGAAATGCCCACAA /<br>GAAAGTAAATACAGGGAACAGAAAGC
Guide RNA A<br>Off-target 2	ATGGGTCACACACCCTCTTC /<br>CATGTTCAGTGCTGAGTACCAA
Guide RNA A<br>Off-target 3	GCTCCAAGTTTCCTGAATAGTTT /<br>TCTTCCGATCCAGTGTTGTG
Guide RNA A<br>Off-target 4	CTACAAGGAACCCCCAAAGG /<br>TGAGCTCTGTGCTCTATGCAG
Guide RNA A<br>Off-target 5	TGTCACCTTTGCCCTTCTTT /<br>CCGCCACATCTGTCTTCC
Guide RNA A<br>Off-target 6	TGAGGCTCTCAAGACACTGC /<br>AGAAACTTGCCAACCGTGCT
Guide RNA A<br>Off-target 7	CATCCCCTGCCAGTCTAAAA /<br>GCACAAGTCTGCAATAAATGG
Guide RNA A<br>Off-target 8	GACAACTAGGAAGCTCTTTTTGG /<br>GGAGGAGATTCAGGACAGCA
Guide RNA A<br>Off-target 9	TCAATTGTGGCAGTGGTTTC /<br>GGGCCCATAGCTGTTTCTTT
Guide RNA A<br>Off-target 10	TGGGGACACGTTAGTGAACA /<br>AGGGCACCAATGATGTGTCT
Guide RNA B<br>Off-target 1	TGGGGAGAGGAATTCATGCA /<br>CCTACAACTGCCGGTCATTG
Guide RNA B<br>Off-target 2	GCACCTCTCAATCCTCTGCT /<br>AGCCCATCCACCAAAAGAAGA
Guide RNA B<br>Off-target 3	CGTCAAATGAGACTGAATCCCTCTA /<br>GCCTCCCATAGTGCTAGGATTAC
Guide RNA B<br>Off-target 4	GGGCGCCACATGTTTGTATT /<br>TGCCCCTCATCATCTCTTGC
Guide RNA B<br>Off-target 5	AAGCCTCTTCCCTGCACTAA /<br>GCAAGGAAGGTGTTGTACTCC
Guide RNA B<br>Off-target 6	ATTTTTCAAGCCCTGTGGTAAC /<br>AGAGACTTTTGCAATGTTCCAGT
Guide RNA B<br>Off-target 7	AGAGGCGCTGCAGTGAATTA /<br>CAGGAACCAAGGGAAAGGCT

Guide RNA B<br>Off-target 8	CTGCCCCAATCCATTTTGCC /<br>TCCTGGGAATGGACTGACCT
Guide RNA B<br>Off-target 9	CCACAGCTGAAAAGCATCCC /<br>TGTTGGTGAGAGCCAGACAC
Guide RNA B<br>Off-target 10	GTGTGGGGACAGATAAGTGGA /<br>CCTTCCTGGGAGCTTGTGTT
**Gene (RT-PCR)**	**Primer**
GAPDH	AGAGGCAGGGATGATGTTCT /<br>TCTGCTGATGCCCCCATGTT
LZTR1 Ex16-17	GTAAAGGAGTCCCACTTCAAC /<br>GCAACAGAGTGATGTCACAG
LZTR1 Ex1	ATGGCTGGACCGGGCAGCA /<br>ACCCACGAACTCGTCGCAGG
**Gene (real-time)**	**Primer**
GAPDH	GGAGCGAGATCCCTCCAAAAT /<br>GGCTGTTGTCATACTTCTCATGG
LZTR1 Ex6-7	GCAACGCCAGGTTGAATGAC /<br>GAGCAGGTGTTCAGTTGGGA
LZTR1 In16-Ex17	GGACATTGGCCCCTTCATTC /<br>TGTGCCTCATGATCAAGCCC
MRAS	CCACCATTGAAGACTCCTACCTG /<br>ACGGAGTAGACGATGAGGAAGC
RIT1	TTCATCAGCCACCGATTCCC /<br>GCAGGCTCATCATCAATACGG
RPL37A	GTGGTTCCTGCATGAAGACAGTG /<br>TTCTGATGGCGGACTTTACCG
TUBB5	CTGGACCGCATCTCTGTGTACT /<br>GCCAAAAGGACCTGAGCGAACA

Table S3: Antibodies used for Western blot, immunocytochemistry and flow cytometry.

**Primary antibody**	**Supplier**	**Resource ID**
α-actinin monoclonal mouse	Sigma-Aldrich	RRID:AB_476766
β-actin monoclonal mouse	Santa Cruz	RRID:AB_1119529
Cas9 polyclonal rabbit	Diagenode	RRID:AB_2715516
HA-Tag monoclonal rabbit	Cell Signaling	RRID:AB_2798368
LZTR1 monoclonal rabbit	Abcam	RRID:AB_3076250
MLC2V polyclonal rabbit	Proteintech	RRID:AB_2147453
MRAS polyclonal rabbit	Proteintech	RRID:AB_10950895
NANOG monoclonal mouse	Thermo Fisher Scientific	RRID:AB_2536677
OCT3/4-PE monoclonal human	Miltenyi Biotec	RRID:AB_2784442
pan-RAS monoclonal mouse	Merck Millipore	RRID:AB_2121151
RIT1 polyclonal rabbit	Abcam	RRID:AB_882379
TRA-1-60-Alexa488 monoclonal mouse	BD Biosciences	RRID:AB_1645379
Vinculin monoclonal mouse	Sigma-Aldrich	RRID:AB_477629
**Secondary antibody**	**Supplier**	**Resource ID**
Alexa488 polyclonal goat anti-rabbit	Thermo Fisher Scientific	RRID:AB_143165
Alexa555 polyclonal donkey anti-mouse	Thermo Fisher Scientific	RRID:AB_2536180
HRP polyclonal donkey anti-rabbit	Sigma-Aldrich	RRID:AB_2722659
HRP polyclonal donkey anti-mouse	Sigma-Aldrich	RRID:AB_772210

Table S4: Plasmids used for virus production.

**Plasmid**	**Supplier**	**Resource ID**
Lenti_SaCRISPR_GFP	Addgene	RRID:Addgene_118636
LentiCRISPRv2GFP	Addgene	RRID:Addgene_82416
pMD2.G	Addgene	RRID:Addgene_12259
psPAX2	Addgene	RRID:Addgene_12260
pAAV-CMV-SauriCas9	Addgene	RRID:Addgene_135964
pAAV-TNNT2-SauriCas9-U6-sgRNA	This study	-
pAAV-TNNT2-SaCas9-U6-sgRNA	This study	-
pAAV-TNNT2-SlugCas9-U6-sgRNA	This study	-
