# Supplementary material for: Preclinical evaluation of CRISPR-based therapies for Noonan syndrome caused by deep-intronic LZTR1 variants
Source: Mol Ther Nucleic Acids. 2024 Jan 23;35(1):102123. doi: 10.1016/j.omtn.2024.102123 (PMC10851011; doi:10.1016/j.omtn.2024.102123)
Supplement: Document S2. Article plus supplemental information [file mmc2.pdf]

Molecular Therapy
Nucleic Acids
Original Article

# Preclinical evaluation of CRISPR-based therapies for Noonan syndrome caused by deep-intronic *LZTR1* variants

Carolin Knauer,[1,2,10] Henrike Haltern,[1,2,10] Eric Schoger,[2,3,4] Sebastian Kügler,[5] Lennart Roos,[1,2,3] Laura C. Zelarayán,[2,3,4,6] Gerd Hasenfuss,[1,2,3] Wolfram-Hubertus Zimmermann,[2,3,4,7,8] Bernd Wollnik,[2,3,9] and Lukas Cyganek[1,2,3,7]

[1]Stem Cell Unit, Clinic for Cardiology and Pneumology, University Medical Center Göttingen, 37075 Göttingen, Germany; [2]DZHK (German Center for Cardiovascular Research), partner site Göttingen, 37075 Göttingen, Germany; [3]Cluster of Excellence "Multiscale Bioimaging: from Molecular Machines to Networks of Excitable Cells" (MBExC), University of Göttingen, 37075 Göttingen, Germany; [4]Institute of Pharmacology and Toxicology, University Medical Center Göttingen, 37075 Göttingen, Germany; [5]Department of Neurology, University Medical Center Göttingen, 37075 Göttingen, Germany; [6]Department of Cardiology and Angiology, University of Giessen, 35390 Giessen, Germany; [7]Fraunhofer Institute for Translational Medicine and Pharmacology ITMP, 37075 Göttingen, Germany; [8]DZNE (German Center for Neurodegenerative Diseases), 37075 Göttingen, Germany; [9]Institute of Human Genetics, University Medical Center Göttingen, 37075 Göttingen, Germany

**Gene variants in *LZTR1* are implicated to cause Noonan syndrome associated with a severe and early-onset hypertrophic cardiomyopathy. Mechanistically, *LZTR1* deficiency results in accumulation of RAS GTPases and, as a consequence, in RAS-MAPK signaling hyperactivity, thereby causing the Noonan syndrome-associated phenotype. Despite its epidemiological relevance, pharmacological as well as invasive therapies remain limited. Here, personalized CRISPR-Cas9 gene therapies might offer a novel alternative for a curative treatment in this patient cohort. In this study, by utilizing a patient-specific screening platform based on iPSC-derived cardiomyocytes from two Noonan syndrome patients, we evaluated different clinically translatable therapeutic approaches using small Cas9 orthologs targeting a deep-intronic *LZTR1* variant to cure the disease-associated molecular pathology. Despite high editing efficiencies in cardiomyocyte cultures transduced with lentivirus or all-in-one adeno-associated viruses, we observed crucial differences in editing outcomes in proliferative iPSCs vs. non-proliferative cardiomyocytes. While editing in iPSCs rescued the phenotype, the same editing approaches did not robustly restore LZTR1 function in cardiomyocytes, indicating critical differences in the activity of DNA double-strand break repair mechanisms between proliferative and non-proliferative cell types and highlighting the importance of cell type-specific screens for testing CRISPR-Cas9 gene therapies.**

## INTRODUCTION

Noonan syndrome (NS) is a congenital multi-systemic disorder that is defined by specific external characteristics accompanied by intellectual and developmental impairment and an increased risk of neoplasia. Further, NS typically presents with a broad variety of heart disease, most commonly pulmonary valve stenosis with or without severe early-onset hypertrophic cardiomyopathy (HCM).[1] With an estimated prevalence of 1 in 1,000–2,500 newborns, it is the second most common syndromic disorder associated with congenital heart defects after Down syndrome.[2] Compared with children with non-syndromic HCM, children with NS-associated HCM are more likely to die from heart failure and are significantly more likely to die at an earlier age.[3,4] Besides the well-known disease-associated genes *PTPN11*, *SOS1*, *RAF1*, *KRAS*, and *RIT1*, autosomal dominant as well as autosomal recessive mutations in leucine zipper like transcription regulator 1 (*LZTR1*) were recently linked to cause NS with severe HCM.[5–7] *LZTR1* encodes an adapter protein of the cullin 3 ubiquitin ligase complex by selectively targeting RAS GTPases as substrates for degradation. In NS, *LZTR1* deficiency results in accumulation of the RAS protein pool and, as a consequence, in RAS-MAPK signaling hyperactivity.[7–9] Despite its epidemiological relevance and ongoing research on NS, pharmacological as well as invasive therapy strategies remain limited as no curative treatment exists.[10]

Recent developments in genome editing using clustered regularly interspaced short palindromic repeats (CRISPR)-Cas9 systems aiming to introduce novel mutations or correct patient-specific mutations have brought about new perspectives on precision medicine and treatment of genetically inherited diseases.[11,12] Alongside several promising clinical studies for potentially fatal diseases based on *ex vivo* genome editing of cells derived from autologous donors,[13] the first phase 1 clinical trials for an *in vivo* application of

Received 1 February 2023; accepted 18 January 2024;
https://doi.org/10.1016/j.omtn.2024.102123.

[10]These authors contributed equally

**Correspondence:** Dr. Lukas Cyganek, Clinic for Cardiology and Pneumology, University Medical Center Göttingen, Robert-Koch-Str. 40, 37075 Göttingen, Germany.
**E-mail:** lukas.cyganek@gwdg.de


This is an open access article under the CC BY license (http://creativecommons.org/licenses/by/4.0/).

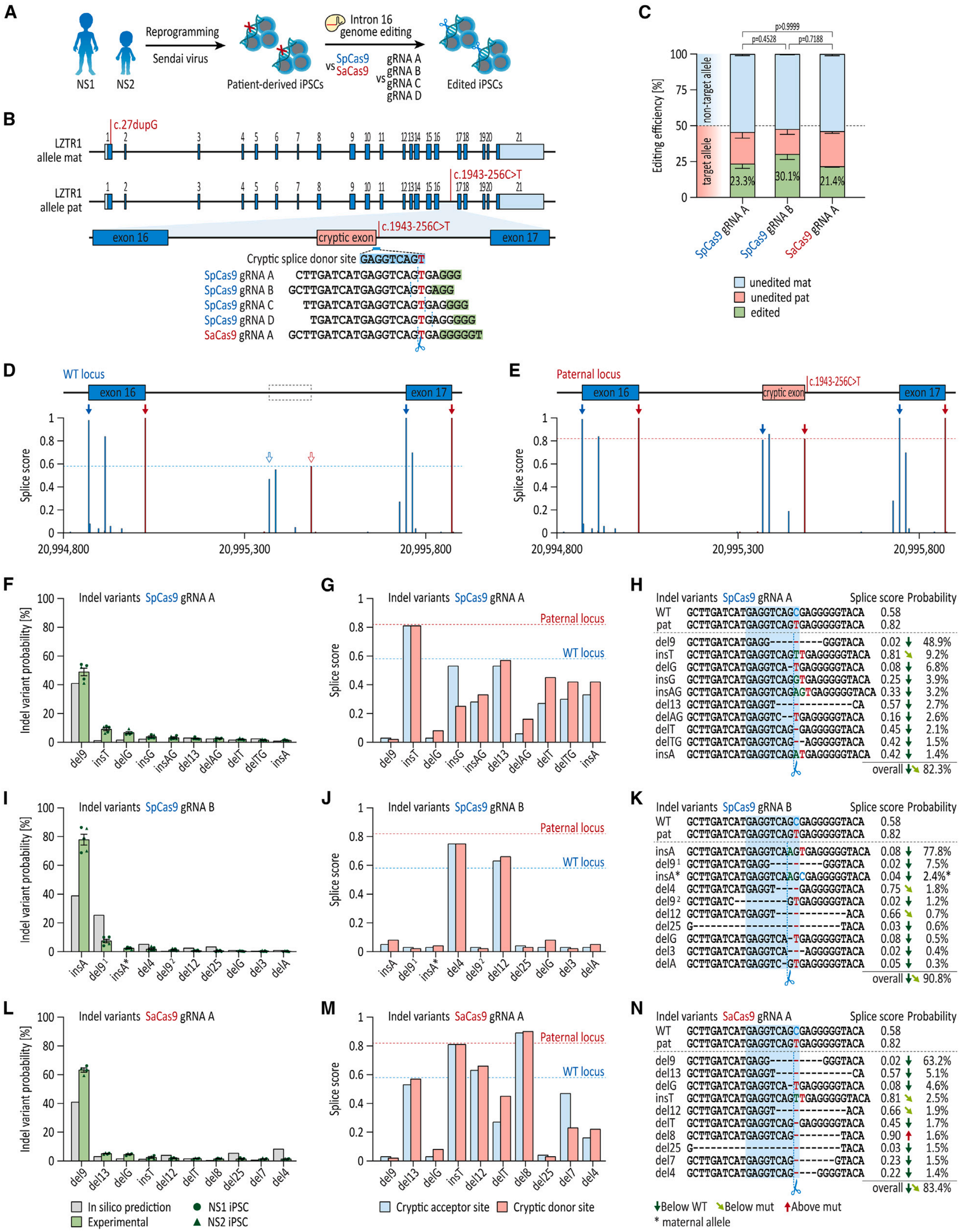


(legend on next page)

CRISPR-Cas9 to treat Leber congenital amaurosis and transthyretin amyloidosis were initiated.[14,15] Further, great translational progress has been made for CRISPR-based treatments of other congenital diseases, such as Duchenne muscular dystrophy and Progeria syndrome,[16–20] proven to be efficient in patient-derived cells as well as mouse and large animal models. It is of great clinical interest to expand these innovative strategies to NS and NS-associated HCM, as CRISPR-based gene therapy might offer curative treatments tailored to the individual patient.

Canonical non-homologous end-joining (cNHEJ) and microhomology-mediated end-joining (MMEJ) are considered the main repair routes of DNA double-strand breaks in both pluripotent and post-mitotic cells such as cardiomyocytes. These were assumed to be inaccurate, random, and unreliable and a major concern for clinical application of CRISPR-based therapies.[21–25] However, recent findings revealed reproducible and even predictable target sequence-specific profiles of insertions and deletions (indels) with cNHEJ and MMEJ upon CRISPR-Cas9 editing.[26–29] As every CRISPR-Cas9 approach differs regarding their genetic locus, potential off-target activity and potential side effects, it is of utmost importance to evaluate each clinically translatable approach *in vitro* and/or *in vivo* for efficacy and safety, ideally in a patient-specific and tissue-specific context.[30] Here, human induced pluripotent stem cell-derived cardiomyocytes (iPSC-CMs) generated from patients with inherited forms of cardiomyopathies are not only suitable to faithfully recapitulate the disease *in vitro*,[31,32] these cells also offer a unique platform to test personalized CRISPR therapies in patient-derived and physiologically relevant cells, by providing a virtually unlimited source of human cells.[16,33]

We recently identified two brothers affected by an autosomal recessive form of NS and severe early-onset HCM due to biallelic *LZTR1*-truncating variants.[7] In a proof-of-concept approach, we demonstrated that CRISPR-Cas9-based genome editing of one of the causative variants was able to rescue the cardiac pathology. Building on these data, in this study we aimed to evaluate different Cas9 orthologs and multiple mutation-specific CRISPR guide RNAs in the established preclinical iPSC-CM models to identify an efficient and safe strategy for a personalized CRISPR-Cas9 gene therapy. We showed that allele-specific editing of the deep-intronic *LZTR1* variant in patient-specific iPSCs resulted in efficient restoration of LZTR1 function and normalization RAS GTPase levels. Further, we applied different small Cas9 orthologs, which allow delivery of the CRISPR-Cas9 components into target cells via a single adeno-associated virus (AAV) genome. Despite high editing efficiencies achieved in AAV-transduced iPSC-CMs, we observed different editing outcomes in iPSC-CMs compared with iPSCs. While editing in iPSCs rescued the phenotype, the same approaches did not robustly restore LZTR1 function in iPSC-CMs, indicating critical differences in the activity of DNA double-strand break repair mechanisms between proliferative iPSCs and non-proliferative iPSC-CMs, thereby highlighting the importance of cell type- and tissue-specific screens for testing suitable CRISPR-Cas9 gene therapies.

## RESULTS

### CRISPR-Cas9-induced indel profiles for LZTR1 intron 16 in patient-specific iPSCs are predictable and reproducible

We have recently identified two brothers who presented with a severe early-onset obstructive HCM, mild facial anomalies, and short stature, resulting in a diagnosis of autosomal recessive NS.[7] A septal myectomy was performed on these patients at the ages of 3.5 years and 6 months, respectively. However, a progressive re-thickening of the ventricular septum after surgery was observed in both siblings, reflecting the need for alternative curative therapies. Whole exome sequencing detected biallelic gene variants in *LZTR1* as causative for the disease: one maternally inherited 1-base pair insertion c.27dupG in the first exon resulting in early frameshift and protein truncation, and one paternally inherited deep-intronic base pair exchange c.1943-256C>T in intron 16 (Figures 1A and 1B). The intronic variant results in introduction of an additional donor splice site leading to the recognition of a cryptic exon between exon 16 and exon 17, and consequently, to the production of a truncated non-functional protein. The *LZTR1* deficiency resulted in the accumulation of RAS GTPases in the patient-specific cells, whereas uni-allelic genetic correction was sufficient to normalize the molecular disease phenotype.[7] We screened the literature for currently described *LZTR1* germline variants causative for NS in either an autosomal recessive or autosomal dominant manner, and searched the gnomAD database for the prevalence of each of the identified NS-associated *LZTR1*

**Figure 1. Predictability and reproducibility of CRISPR-Cas9-induced indel profiles for *LZTR1* intron 16 in patient-specific iPSCs**

(A) Generation of patient-specific iPSCs from two NS patients by reprogramming of patient skin fibroblasts via integration-free Sendai virus for evaluation of CRISPR-based gene therapies. (B) Depiction of the genome editing approach for allele-specific targeting of the deep-intronic variant in *LZTR1* intron 16 by CRISPR-Cas9 using different guide RNA and Cas9 combinations. (C) Quantification of editing efficiencies for SpCas9 with guide RNA A and guide RNA B and SaCas9 with guide RNA A using amplicon sequencing 3–4 days post-transfection; n = 5 individual approaches from two iPSC lines. (D and E) *In silico*-based splice site predictions of the WT locus (D) and the mutated paternal locus (E). (F) Comparison of indel variants for SpCas9 and guide RNA A in transfected bulks, assessed by amplicon sequencing, with computational indel prediction. (G) Splice score prediction of the cryptic acceptor and donor sites for the top 10 indel variants generated by SpCas9 and guide RNA A. (H) Analysis of indel variant probabilities generated by SpCas9 and guide RNA A and computational prediction of splice site motifs. (I) Comparison of indel variants for SpCas9 and guide RNA B in transfected bulks, assessed by amplicon sequencing, with computational indel prediction. (J) Splice score prediction of the cryptic acceptor and donor sites for the top 10 indel variants generated by SpCas9 and guide RNA B. (K) Analysis of indel variant probabilities generated by SpCas9 and guide RNA B and computational prediction of splice site motifs. (L) Comparison of indel variants for SaCas9 and guide RNA A in transfected bulks, assessed by amplicon sequencing, with computational indel prediction. (M) Splice score prediction of the cryptic acceptor and donor sites for the top 10 indel variants generated by SaCas9 and guide RNA A. (N) Analysis of indel variant probabilities generated by SaCas9 and guide RNA A and computational prediction of splice site motifs. Data were analyzed by nonparametric Kruskal-Wallis test with Dunn's correction and are presented as mean ± SEM (C).

variants in the entire population (Table 1). This analysis revealed that the intronic variant c.1943-256C>T is one of the most commonly occurring *LZTR1* variants with a high prevalence of up to 1 in 10,000 individuals. Hence, this intronic variant is a therapeutically relevant target for a sustainable CRISPR-based gene therapy approach.

We postulated that cNHEJ- and MMEJ-induced indels in close proximity to the deep-intronic variant would cause disruption of the cryptic donor splice site without compromising regular splicing of the *LZTR1* transcript, thereby reintroducing regular translation upon editing of the *LZTR1* allele. Patient-specific iPSCs from both NS patients were utilized for a preclinical CRISPR screen, in order to identify a suitable CRISPR-Cas9 system regarding efficiency and safety for clinical translation (Figure 1A). For the widely used Cas9 ortholog *Streptococcus pyogenes* (SpCas9) with the protospacer adjacent motif *5′-NGG-3′*, we evaluated four different CRISPR guide RNAs (guide RNA A, B, C, and D), all allowing an allele-specific targeting of the mutated *LZTR1* intron 16 (Figure 1B). In addition, we included the compact Cas9 ortholog *Staphylococcus aureus* (SaCas9) in our screen. Despite its comparatively more complex canonical protospacer adjacent motif *5′-NNGRRT-3′*, one CRISPR guide RNA (guide RNA A) met the requirements to be used in combination with the SaCas9 for allele-specific editing. Patient-specific iPSCs were transfected with the different Cas9 and guide RNA combinations using a ribonucleoprotein-based approach. Initial evaluation of the transfected bulk 3–4 days post-transfection by Sanger sequencing revealed efficient on-target editing by SpCas9 in combination with guide RNA A and B (Figure S1). Further, robust editing was observed for SaCas9 with guide RNA A. In contrast, transfection of guide RNA C and D resulted in lower overall editing efficiencies; hence, these two guide RNAs were excluded from further analysis. Subsequently, amplicon sequencing was performed to quantify editing efficiencies as well as to discriminate the precision of the different CRISPR-Cas9 approaches to selectively target the mutated intron 16 allele. In agreement with the Sanger sequencing data, both guide RNAs for SpCas9 as well as the SaCas9 application expressed high editing efficiencies with indel frequencies ranging between 21% and 30% out of all sequenced alleles (Figure 1C). Moreover, more than 50% of all alleles in each approach were of maternal origin (non-target allele) and remained unedited at the intron 16 locus, indicating that both SpCas9 and SaCas9 were highly selective for the mutated allele. Considering that the targeted allele accounts for half of each diploid cell's genotype, editing efficiencies of 21%–30% at the paternal allele would, in principle, imply that 42%–60% of the cells were genetically modified.

However, high editing efficiencies at the cryptic donor splice site do not necessarily translate to a beneficial effect on pathophysiological splicing mechanisms. It remains crucial to prove whether the cNHEJ- and MMEJ-induced changes at the target site reduce the likelihood to serve as a cryptic splice site (Figures 1D and 1E). We anticipated that every individual CRISPR-Cas9 approach would have a tendency to induce certain indels more frequently than others. In-depth analysis of all edited sequences revealed that dependent on the precise location of the Cas9-induced double-strand break, which in turn is influenced by the respective guide RNAs applied, unique indel patterns for each CRISPR-Cas9 complex were created (Figures 1F–1N). Importantly, the CRISPR editing repeatedly generated the same indels at almost identical frequencies resulting in highly reproducible indel profiles. Moreover, computational predictions of indel events by the web-based tool inDelphi[28] were consistent with the experimentally determined profiles (Figures 1F, 1I, and 1L). The top indel variants generated by each CRISPR-Cas9 approach were all predicted to reduce the likelihood of splicing the cryptic exon,[45] with most indels showing even lower splice scores than the wild-type (WT) allele (Figures 1G, 1J, and 1M). The comparison of the indel profiles generated by the tested guide RNA and Cas9 combinations uncovered several findings. First, each indel pattern was dominated by a highly frequently occurring top variant: a 9-base pair deletion for guide RNA A, with 49% and 63% for SpCas9 and SaCas9, respectively, or a 1-base pair insertion of an adenosine in 78% of editing outcomes for SpCas9 with guide RNA B (Figures 1H, 1K, and 1N). Second, despite their cleavage sites being directly adjacent to one another, indel profiles varied greatly between guide RNA A and guide RNA B. At the same time, SpCas9 and SaCas9 induced identical top variants when guided by guide RNA A; indels of lower frequencies, however, differed between the two enzymes. Importantly, based on *in silico* splicing predictions,[45] most indel variants (between 82% and 91% of indels) displayed a reduced probability to be recognized as splice site compared with the pathological C to T mutation.

In addition to assessing on-target editing, we aimed to investigate the specificity of the different CRISPR-Cas9 combinations by analyzing potential off-target editing of the non-mutated intron 16 locus. Here, WT iPSCs served as an instrument to evaluate editing events on the non-targeted allele. Although we detected a few missense and indel variants at the *LZTR1* intron 16 locus for all three CRISPR-Cas9 approaches, the overall off-target editing in WT cells was very low (Figure S2). Further, the majority of the detected genetic alternations (except for c.1943-256C>T) were not predicted to introduce a cryptic splice site.

Collectively, our data demonstrated that genome editing with both SpCas9 and SaCas9 in patient-specific proliferative iPSCs enables selective targeting of the mutated *LZTR1* intron 16 by inducing highly reproducible indel profiles, predicted to restore regular splicing of the *LZTR1* transcript.

### CRISPR-Cas9-induced indels at LZTR1 intron 16 restore LZTR1 function and normalize RAS GTPase levels

Motivated by the initial experimental and computational data, we aimed to evaluate the molecular consequences of the most frequently generated indels at the *LZTR1* intron 16 for the splicing machinery and for functionality of the LZTR1-cullin 3 ubiquitin ligase complex. The transfected iPSCs from both NS patients were singularized and individual CRISPR-edited iPSC clones harboring the top indel variants for guide RNA A (9-base pair deletion, del9) and for guide RNA B (1-base pair insertion of adenosine, insA) were established (Figure S3). The patient-derived and CRISPR-edited iPSCs were verified for pluripotency. Further, molecular karyotyping of the edited

**Table 1. Prevalence of *LZTR1* germline variants causative for NS**

| Variant genomic | Variant protein | NS form | If biallelic: 2ND variant | gnomAD exome v2 | gnomAD genome v2 | gnomAD genome v3 | gnomAD mean | Reference |
|---|---|---|---|---|---|---|---|---|
| c.1407G>A | p.W469* | recessive | p.Y749C | 0,000138 | 0,000223 | 0,0001708 | 0,00017727 | Pagnamenta et al.[34] |
| c.1943-256C>T | | recessive | p.Q10Afs*, p.Y726*, p.R688G, c.2325+G>A, c.1943-256C>T | 0,00004073 | 0,0001597 | 0,00007911 | 0,00009318 | Johnston et al., Hanses et al.[6,7] |
| c.628C>T | p.R210* | recessive | p.D531N, p.V579M, c.2220-17C>A | 0,00007195 | 0,00009567 | 0,00009873 | 0,00008878 | Johnston et al., Pagnamenta et al.[6,34] |
| c.2062C>T | p.R688C | recessive | c.1149 + 1G>T | 0,00004431 | 0,00009575 | 0,0000526 | 0,00006422 | Pagnamenta et al.[34] |
| c.1084C>T | p.R362* | recessive | c.1149 + 1G>T | 0,00005975 | 0,00006378 | 0,00006572 | 0,00006308 | Perin et al.[35] |
| c.27dupG | p.Q10Afs* | recessive | c.1943-256C>T | 0,0001004 | 0,00003187 | 0,000046 | 0,00005942 | Hanses et al.[7] |
| c.27delG | p.Q10Rfs* | recessive | c.1149+G>A | 0,00006274 | not detected | 0,00001972 | 0,00002749 | Johnston et al.[6] |
| c.1385T>C | p.I462T | recessive | p.A461D | 0,00001613 | 0,00003187 | 0,00001314 | 0,00002038 | Pagnamenta et al.[34] |
| c.1591G>A | p.D531N | recessive | p.R210* | 0,00002566 | not detected | 0,00003285 | 0,00001950 | Pagnamenta et al.[34] |
| c.2462T>C | p.I821T | recessive | p.I821T | not detected | 0,00003187 | 0,00001314 | 0,00001500 | Johnston et al.[6] |
| c.2090G>A | p.R697Q | recessive | c.2407-2A>G | 0,00002388 | not detected | 0,00001972 | 0,00001453 | Johnston et al.[6] |
| c.2220-17C>A | | recessive | p.R210* | 0,000003994 | 0,00003189 | not detected | 0,00001196 | Johnston et al.[6] |
| c.1735G>A | p.V579M | recessive | p.R210*, c.2070-2A>G | 0,00001998 | not detected | 0,00001314 | 0,00001104 | Pagnamenta et al., Perin et al.[34,35] |
| c.508C>T | p.R170W | recessive | p.I205T | 0,00001195 | not detected | 0,00001972 | 0,00001056 | Johnston et al.[6] |
| c.1964T>C | p.M655T | recessive | p.M655T | 0,00001194 | not detected | 0,00001972 | 0,00001055 | our clinic |
| c.-38T>A | | recessive | p.W437* | 0,000008025 | not detected | 0,00001315 | 0,00000706 | Pagnamenta et al.[34] |
| c.2246A>G | p.Y749C | recessive | p.W469* | 0,000003981 | not detected | 0,00001318 | 0,00000572 | Pagnamenta et al.[34] |
| c.1149 + 1G>A | | recessive | p.Q10fs* | 0,000008114 | not detected | 0,000006571 | 0,00000490 | Johnston et al.[6] |
| c.2407-2A>G | | recessive | p.R697Q | not detected | not detected | 0,00001315 | 0,00000438 | Johnston et al.[6] |
| c.2325 + 1G>A | | recessive | c.1943-256C>T | not detected | not detected | 0,00001314 | 0,00000438 | Johnston et al.[6] |
| c.2074T>A | p.F692L | recessive | p.F692L | 0,00000398 | not detected | 0,000006571 | 0,00000352 | Güemes et al.[36] |
| c.1430C>T | p.A477V | dominant | | 0,000008276 | not detected | not detected | 0,00000276 | Ferrari et al.[37] |
| c.614T>C | p.I205T | recessive | p.R170W | 0,000007978 | not detected | not detected | 0,00000266 | Johnston et al.[6] |
| c.850C>T | p.R284C | dominant | | not detected | not detected | 0,000006572 | 0,00000219 | Yamamoto et al., Jacquinet et al.[38,39] |
| c.848G>A | p.R283Q | dominant | | not detected | not detected | 0,000006571 | 0,00000219 | Umeki et al.[40] |
| c.2102C>A | p.P701H | recessive | c.2069 + 2T>C | not detected | not detected | 0,000006571 | 0,00000219 | Umeki et al.[40] |
| c.1311G>A | p.W437* | recessive | c.-38T>A | not detected | not detected | 0,000006569 | 0,00000219 | Pagnamenta et al.[34] |
| c.1149 + 1G>T | | recessive | p.R362*, p.R688C | 0,000004057 | not detected | not detected | 0,00000135 | Pagnamenta et al., Perin et al.[34,35] |
| c.1687G>C | p.E563Q | recessive | p.E563Q | 0,000004 | not detected | not detected | 0,00000133 | Johnston et al.[6] |
| c.2178C>A | p.Y726* | recessive | c.1943-256C>T | 0,000003986 | not detected | not detected | 0,00000133 | Johnston et al.[6] |
| c.2264G>A | p.R755Q | recessive | p.H121D | 0,000003982 | not detected | not detected | 0,00000133 | Johnston et al.[6] |

(*Continued on next page*)

**Table 1. *Continued***

| Variant genomic | Variant protein | NS form | If biallelic: 2ND variant | gnomAD exome v2 | gnomAD genome v2 | gnomAD genome v3 | gnomAD mean | Reference |
|---|---|---|---|---|---|---|---|---|
| c.2070-2A>G | | recessive | p.V579M | 0,000003981 | not detected | not detected | 0,00000133 | Perin et al.[35] |
| c.290G>T | p.R97L | dominant | | not detected | not detected | not detected | – | Pagnamenta et al.[34] |
| c.347C>T | p.A116V | dominant | | not detected | not detected | not detected | – | Ghedira et al..[41] |
| c.355T>C | p.Y119H | dominant | | not detected | not detected | not detected | – | Ferrari et al.[37] |
| c.356A>G | p.Y119C | dominant | | not detected | not detected | not detected | – | Yamamoto et al.[38] |
| c.361C>G | p.H121D | recessive | p.R755Q | not detected | not detected | not detected | – | Johnston et al.[6] |
| c.406T>C | p.Y136H | dominant | | not detected | not detected | not detected | – | Pagnamenta et al.[34] |
| c.407A>G | p.Y136C | dominant | | not detected | not detected | not detected | – | Pagnamenta et al.[34] |
| c.428A>G | p.N143S | dominant | | not detected | not detected | not detected | – | Umeki et al.[40] |
| c.434A>T | p.N145I | dominant | | not detected | not detected | not detected | – | Pagnamenta et al.[34] |
| c.509G>C | p.R170P | recessive | p.C792G | not detected | not detected | not detected | – | Chen et al.[42] |
| c.606_650del | p.M202fs* | dominant | | not detected | not detected | not detected | – | Umeki et al.[40] |
| c.650A>C | p.E217A | recessive | p.E217A | not detected | not detected | not detected | – | Johnston et al.[6] |
| c.730T>C | p.S244P | dominant | | not detected | not detected | not detected | – | Güemes et al.[36] |
| c.731C>G | p.S244C | dominant | | not detected | not detected | not detected | – | Pagnamenta et al.[34] |
| c.740G>A | p.S247N | dominant | | not detected | not detected | not detected | – | Yamamoto et al.[38] |
| c.742G>A | p.G248R | dominant | | not detected | not detected | not detected | – | Pagnamenta et al., Güemes et al., Yamamoto et al., Umeki et al.[34,36,38,40] |
| c.743G>A | p.G248E | dominant | | not detected | not detected | not detected | – | Farncombe et al.[43] |
| c.756_758del | p.N253del | dominant | | not detected | not detected | not detected | – | Umeki et al.[40] |
| c.859C>T | p.H287Y | dominant | | not detected | not detected | not detected | – | Yamamoto et al.[38] |
| c.1382C>A | p.A461D | recessive | p.I462T | not detected | not detected | not detected | – | Pagnamenta et al.[34] |
| c.1660G>C | p.A554P | dominant | | not detected | not detected | not detected | – | Umeki et al.[40] |
| c.1739T>C | p.L580P | recessive | p.L580P | not detected | not detected | not detected | – | Busley et al.[44] |
| c.2062C>G | p.R688G | recessive | c.1943-256C>T | not detected | not detected | not detected | – | Johnston et al.[6] |
| c.2069 + 2T>C | | recessive | p.P701H | not detected | not detected | not detected | – | Umeki et al.[40] |
| c.2374T>G | p.C792G | recessive | p.R170P | not detected | not detected | not detected | – | Chen et al.[42] |

For gnomAD exome v2: around 250,000 alleles in total; for gnomAD genome v2: around 31,000 alleles in total; for gnomAD genome v3: around 152,000 alleles in total; for calculation of gnomAD mean frequencies: not detected = 0

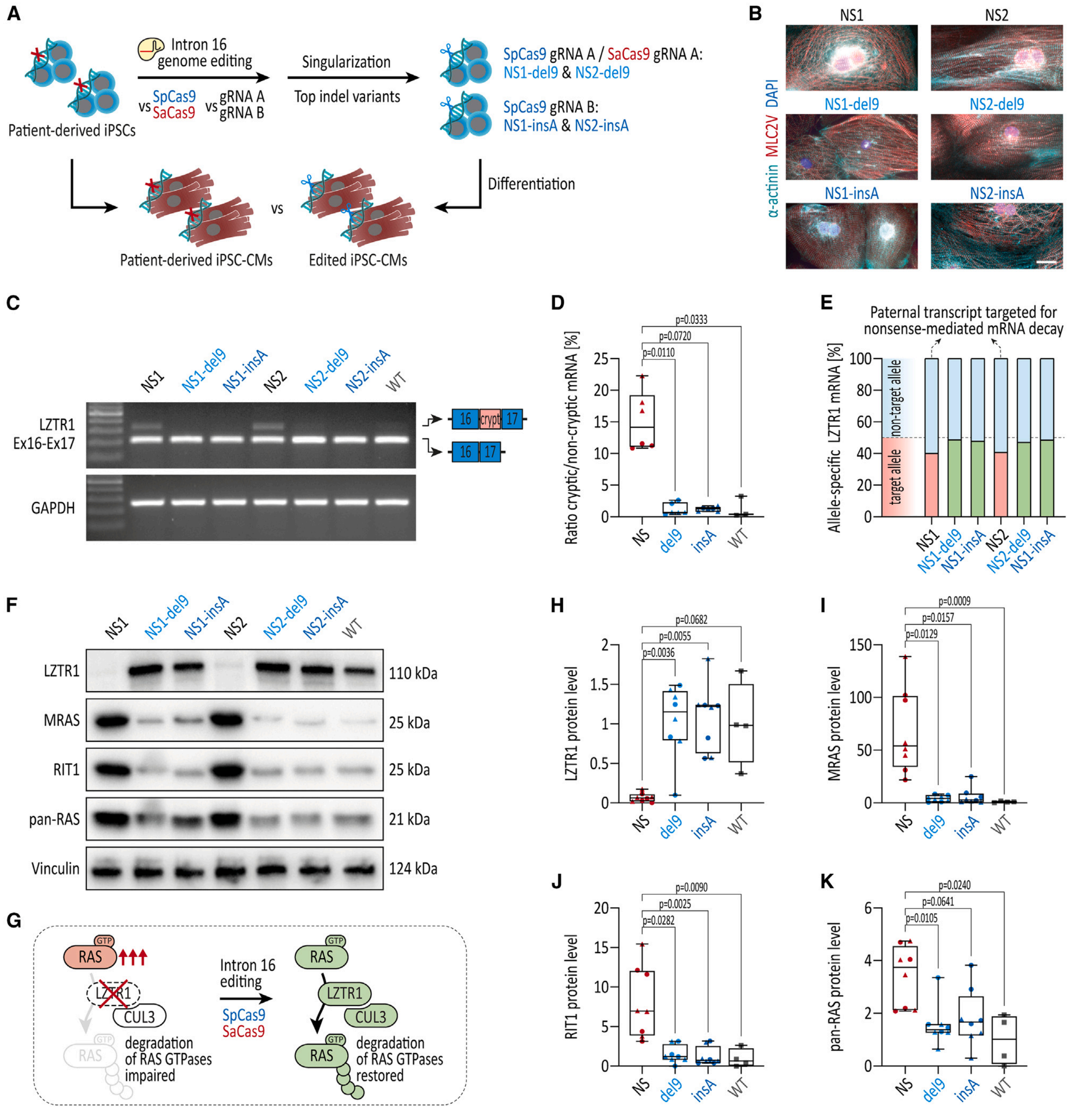


**Figure 2. Restoration of LZTR1 function and normalization of RAS GTPase levels upon CRISPR-Cas9 editing of *LZTR1* intron 16 in patient-specific iPSCs**

(A) CRISPR-edited iPSCs and unedited patient-specific iPSCs were differentiated into ventricular iPSC-CMs and analyzed for LZTR1 restoration and accumulation of RAS levels as indicator of LZTR1 function at day 30 of differentiation. (B) Representative images of iPSC-CMs stained for α-actinin and ventricular-specific MLC2V indicated a regular and well-organized sarcomeric assembly across all iPSC lines; scale bar, 20 μm. (C) Evaluation of regular splicing of the *LZTR1* transcript, assessed by reverse-transcriptase PCR, revealed exclusion of the cryptic exon between exons 16 and 17 in CRISPR-edited iPSC-CMs in comparison with unedited iPSC-CMs from the patients. Analysis of GAPDH expression served as control. (D) Quantification of ratios for abundance of the cryptic exon vs. the non-cryptic variant revealed significant reduction of the cryptic variant in CRISPR-edited iPSC lines; n = 3 individual differentiations per iPSC line. (E) Evaluation of maternal and paternal mRNA transcript expression, assessed by amplicon sequencing, showed escape of the paternal transcript from nonsense-mediated mRNA decay in CRISPR-corrected iPSC-CMs compared with patient-specific iPSC-CMs at day 30 of differentiation. (F) Representative blots of LZTR1, MRAS, RIT1, and pan-RAS (recognizing HRAS, KRAS, and NRAS), assessed by western blot, revealed normalization of RAS GTPase levels in CRISPR-edited iPSC-CMs in comparison with unedited iPSC-CMs from the patients; Vinculin served as loading control. (G)

*(legend continued on next page)*

iPSC clones confirmed chromosomal stability after genome editing and passaging. No potential off-target modifications were detected in the established iPSC lines in the 10 most likely off-target sites (Figure S4).

Recent findings by our group and others demonstrated that *LZTR1* deficiency resulted in strong accumulation of the RAS GTPase protein pool in cardiomyocytes.[7,44,46] Accordingly, patient-specific, CRISPR-edited, as well as WT iPSC lines were differentiated into functional ventricular-like iPSC-CMs in feeder-free culture conditions,[47] and were subjected to molecular phenotyping on day 30 of differentiation (Figure 2A). Immunocytochemical staining of myocardium-specific proteins revealed a well-organized sarcomeric structure in the patient-specific iPSC-CMs as well as the edited iPSC-CMs with a pronounced striated expression of α-actinin and ventricular-specific MLC2V (Figure 2B). To explore the impact of the top indel variants del9 and insA on splicing, iPSC-CMs were analyzed at the transcriptional level (Figures 2C and 2D). Although the *LZTR1* transcript from the paternal allele is targeted for nonsense-mediated decay, incorporation of the cryptic exon between exons 16 and 17 was robustly detected in both patient-specific iPSC-CMs. In contrast, and in line with a reduced splice site probability, a loss of the cryptic exon was observed in all CRISPR-edited cell lines, suggesting restoration of the physiological *LZTR1* transcript splicing upon CRISPR-Cas9 editing (Figures 2C and 2D). Amplicon sequencing of the *LZTR1* transcript flanking exon 1, which allows allele-specific discrimination of the maternal and paternal transcripts, revealed that the paternal transcript harboring the del9 and insA indel variants is no longer targeted for nonsense-mediated mRNA decay (Figure 2E).

To further clarify whether the splicing-corrected *LZTR1* mRNA translates to a functional protein in the CRISPR-edited iPSC-CMs, we analyzed endogenous LZTR1 and RAS GTPase protein levels as a direct indicator of LZTR1 function (Figures 2F and 2G). While no LZTR1 was detected in the samples from both NS patients, uniallelic editing of the paternal allele was sufficient to restore LZTR1 protein levels to WT levels (Figures 2F and 2H). As anticipated, western blot analyses showed strong accumulation of the muscle RAS homolog MRAS, RIT1, and, to a lower extent, the classical RAS proteins (HRAS, KRAS, and NRAS; detected by pan-RAS) in the patient-specific *LZTR1*-deficient iPSC-CM cultures (Figures 2F–2K). In contrast, similar mRNA expression levels of *LZTR1* and the different RAS proteins in the patient-specific and CRISPR-edited iPSC-CMs were observed, indicating a post-translational cause for the high RAS protein levels (Figure S5). Strikingly, CRISPR-edited iPSC-CMs harboring the most frequently occurring indel variants upon CRISPR editing with guide RNA A and guide RNA B, del9 and insA, respectively, normalized the abundance of RAS GTPases to WT levels, confirming that correction of one *LZTR1* allele is sufficient to regulate the protein pool of RAS GTPases in cardiomyocytes (Figures 2F–2K). iPSC-CM cultures from both parents, each with only one functional copy of *LZTR1*, confirmed that mono-allelic expression of LZTR1 is able to adequately regulate the abundance of RAS proteins in cells (Figure S6).

Although the monoclonal analysis for the top indel variants might represent around 50%–80% of edited cells upon CRISPR-based treatment, application of CRISPR-Cas9 generates a mixture of indel variants, including non-transfected, unedited cells. For this reason, we decided to omit the selection process and generated iPSC-CMs from transfected bulk containing all indel variants at the respective frequencies as well as unedited cells, thereby more closely mimicking a translational scenario. Here, the CRISPR-treated iPSC-CMs with both SpCas9 and SaCas9 showed a substantial reduction of MRAS and RIT1 protein levels compared with the untreated patient-specific iPSC-CMs, verifying the translational potential of the CRISPR-based therapeutic approach (Figure S7).

Taken together, these data revealed that allele-specific editing of the mutated *LZTR1* intron 16 with both SpCas9 and SaCas9 in proliferative iPSCs is able to correct physiological splicing, to restore LZTR1 function, and to normalize the pool of RAS GTPases.

### CRISPR-Cas9 genome editing in non-proliferative iPSC-CMs generates distinct indel profiles compared with proliferative iPSCs

Considering that CRISPR-based gene therapies for NS-associated HCM require robust editing in cardiac tissue, we evaluated CRISPR-Cas9 genome editing in differentiated patient-specific iPSC-CMs. To recapitulate the editing approaches successfully applied at the iPSC level, SpCas9 in combination with guide RNA A or B and SaCas9 in combination with guide RNA A were delivered to patient-specific iPSC-CMs via lentiviral transduction (Figures 3A and 3B). To monitor Cas9 expression in transduced iPSC-CMs, a GFP reporter was co-expressed via a 2A self-cleaving peptide in the lentiviral vectors. After 28 days of lentiviral treatment, robust GFP expression was detected in iPSC-CM cultures transduced with the different Cas9 orthologs without obvious variations in transduction rates (Figure 3C). In contrast, deep amplicon sequencing revealed major differences in editing efficiency between the different lentiviral vectors, ranging from 4% for SpCas9 with guide RNA A to 29% for SaCas9 with guide RNA A (Figures 3D, 3F, and 3H). Furthermore, editing using guide RNA A with either SpCas9 or SaCas9 in iPSC-CMs resulted in a distinct indel profile compared with iPSCs: while a 9-base pair deletion was the most common variant in iPSCs for both Cas9 orthologs, editing at the iPSC-CM level was dominated by a 1-base pair insertion of a thymidine (insT), particularly for SaCas9 with 29% insT vs. 8% del9 (Figures 3E and 3I). Based on *in silico*

Model of *LZTR1*-mediated regulation of RAS-MAPK signaling: *LZTR1* deficiency causes accumulation of RAS GTPases and hyperactivity of the signaling pathway; CRISPR-Cas9-based gene therapy targeting *LZTR1* intron 16 restores LZTR1 function, thereby normalizing RAS-MAPK signaling. (H–K) Quantitative analysis of western blots for LZTR1 (H), MRAS (I), RIT1 (J), and pan-RAS (K); data were normalized to total protein and to the corresponding WT samples on each membrane; n = 4 independent differentiations per iPSC line. Data were analyzed by nonparametric Kruskal-Wallis test with Dunn's correction and are presented as mean ± SEM (D, H, I, J, K).

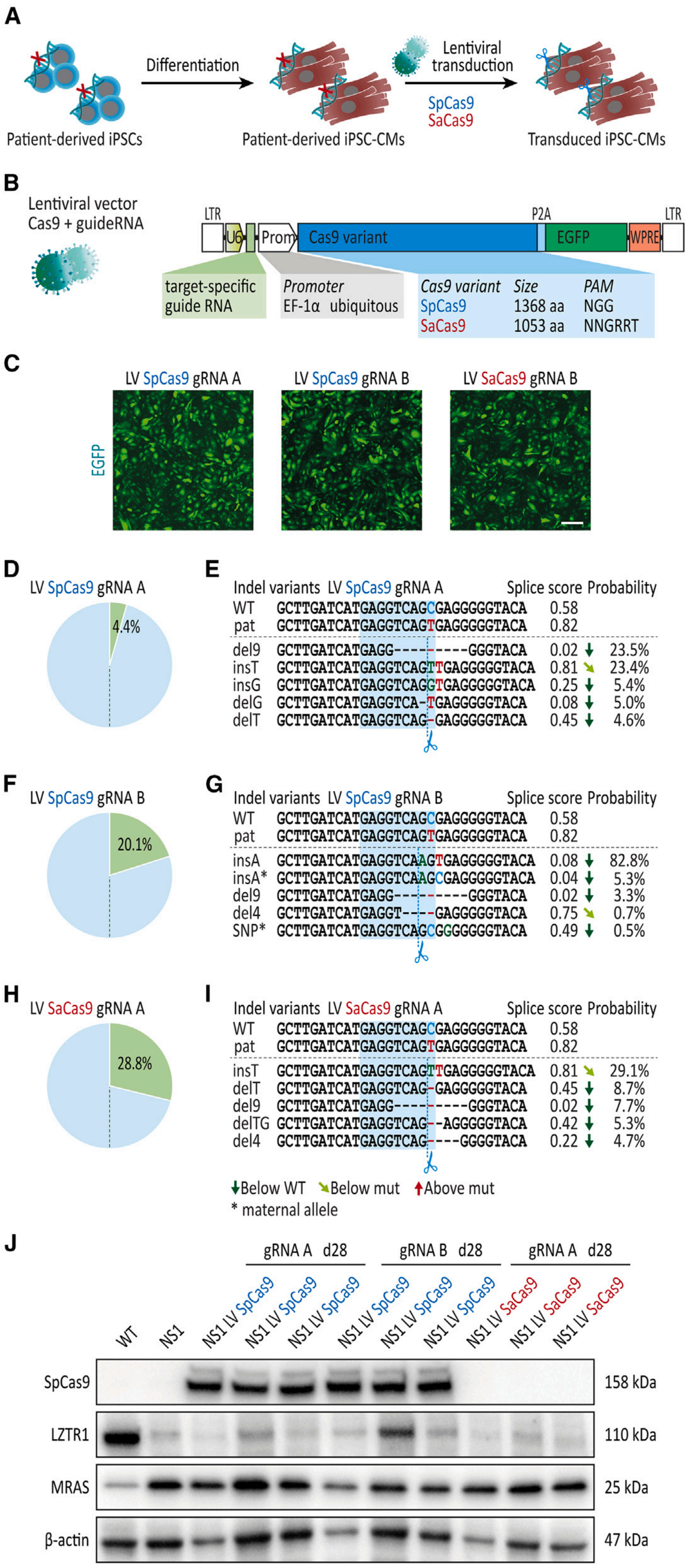


**Figure 3. Distinct indel profiles upon CRISPR-Cas9 editing of *LZTR1* intron 16 in patient-specific iPSC-CMs compared with iPSCs**

(A) Patient-specific iPSC-CMs at day 30 of differentiation were transduced with lentivirus encoding SpCas9 and SaCas9 for evaluation CRISPR-Cas9 gene editing in differentiated iPSC-CMs 4 weeks post-transduction; n = 3 independent transductions per lentiviral construct. (B) Schematic presentation of lentiviral vectors encoding Cas9 and EGFP under control of the *EF-1α* promoter together with a guide RNA expression cassette. (C) Representative images of iPSC-CMs 4 weeks post-transduction showing robust expression of delivered genes across all lentiviral vectors; scale bar, 200 μm. (D) Analysis of editing efficiency, assessed by amplicon sequencing, in patient-specific iPSC-CMs transduced with SpCas9 and guide RNA A. (E) Analysis of indel variant probabilities generated by SpCas9 and guide RNA A and computational prediction of splice site motifs in transduced iPSC-CMs. (F) Analysis of editing efficiency, assessed by amplicon sequencing, in patient-specific iPSC-CMs transduced with SpCas9 and guide RNA B. (G) Analysis of indel variant probabilities generated by SpCas9 and guide RNA B and computational prediction of splice site motifs in transduced iPSC-CMs. (H) Analysis of editing efficiency, assessed by amplicon sequencing, in patient-specific iPSC-CMs transduced with SaCas9 and guide RNA A. (I) Analysis of indel variant probabilities generated by SaCas9 and guide RNA A and computational prediction of splice site motifs in transduced iPSC-CMs. (J) Representative blots of SpCas9, LZTR1, and MRAS levels, assessed by western blot, showed no apparent restoration of LZTR1 function in patient-specific iPSC-CMs 4 weeks post-transduction in comparison with unedited iPSC-CMs from the patient; β-actin served as loading control.

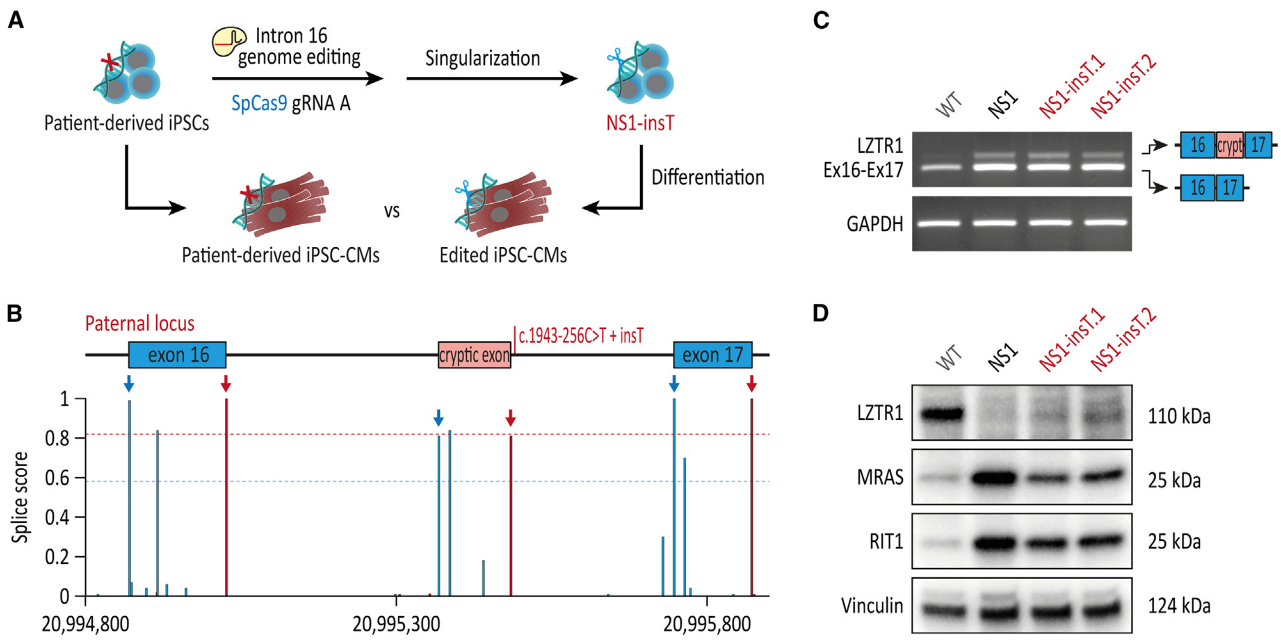


**Figure 4. No restoration of LZTR1 function in patient-specific iPSCs with indel variant insT**

(A) Generation of monoclonal CRISPR-edited iPSCs harboring the frequent indel variant insT by singularization of patient-specific iPSCs transfected with SpCas9 and guide RNA A. (B) *In silico*-based splice site prediction of the mutated paternal locus harboring the frequent indel variant insT. (C) Evaluation of regular splicing of the *LZTR1* transcript, assessed by reverse-transcriptase PCR, revealed no differences between iPSC-CMs harboring the indel variant insT and the unedited iPSC-CMs from the patient. Analysis of GAPDH expression served as control. (D) Representative blots of LZTR1, MRAS, and RIT1, assessed by western blot, revealed no restoration of LZTR1 function in iPSC-CMs harboring the indel variant insT in comparison with unedited iPSC-CMs from the patient; Vinculin served as loading control.

predictions, the insT indel displayed a similar probability of being detected as a cryptic splice site compared with the pathological c.1943-256C>T variant. In contrary, SpCas9 editing with guide RNA B confirmed insA as the predominant indel variant in iPSC-CMs with 83% of editing outcomes, consistent with the experiments in patient-specific iPSCs (Figure 3G).

To prove whether editing after lentiviral treatment leads to a rescue of protein function, endogenous LZTR1 and RAS GTPase levels were analyzed by western blot in transduced iPSC-CMs. First, as expected from the low editing rate of the SpCas9 with guide RNA A, no LZTR1 protein and no reduction in MRAS or RIT1 levels were detected in treated iPSC-CM cultures (Figure 3J). Second, although most of the cells edited by SpCas9 with guide RNA B were corrected by the beneficial insA indel, the presence of LZTR1 was abundant in only one out of three transduced cultures. Consistent with this, no apparent reduction in RAS GTPase levels was observed (Figure 3J). Third, the high editing efficiency for SaCas9 with guide RNA A did not correlate with LZTR1 expression, suggesting that the top indel variant insT may not be able to correct intron 16 splicing of the mutated *LZTR1* intron 16 allele (Figure 3J).

To further investigate these results, we aimed to evaluate the influence of the most frequent indel variant insT induced by guide RNA A in iPSC-CMs on splicing and LZTR1 function. To this end, we generated isogenic iPSC clones harboring the insT variant at the paternal allele of *LZTR1* intron 16 (Figure 4A). In accordance with the high splice site probability for insT based on *in silico* predictions (Figure 4B), no loss of the cryptic exon was observed in the CRISPR-edited iPSC-CMs harboring the thymidine insertion (Figure 4C). Western blot analyses showed no restoration of LZTR1 proteins and consequently a similarly high accumulation of MRAS and RIT1 in the CRISPR-edited iPSC-CMs harboring the insT indel compared with the pathological c.1943-256C>T variant (Figure 4D).

In summary, these data revealed that allele-specific editing of the mutated *LZTR1* intron 16 in non-proliferative iPSC-CMs does not necessarily generate similar indel profiles compared with proliferative iPSCs. While editing in combination with guide RNA B generated the same top indel variant in iPSC-CMs compared with iPSCs, editing in combination with guide RNA A generated a distinct indel profile with the top variant insT being unable to restore LZTR1 function and to normalize the pool of RAS GTPases in non-proliferative iPSC-CMs.

### All-in-one AAVs enable efficient CRISPR-Cas9 genome editing in non-proliferative iPSC-CMs

AAV-based gene therapies allow selective targeting of defined cell types and organs (based on the AAV serotype and promoter applied) and have been proven to be efficient in targeting cardiac tissue with an acceptable safety profile.[48,49] Here, the limited capacity of AAVs (typically below 5 kb) precludes the design of vectors harboring both the common SpCas9 and the locus-specific guide RNA cassette in a single all-in-one AAV construct. We designed an all-in-one AAV vector

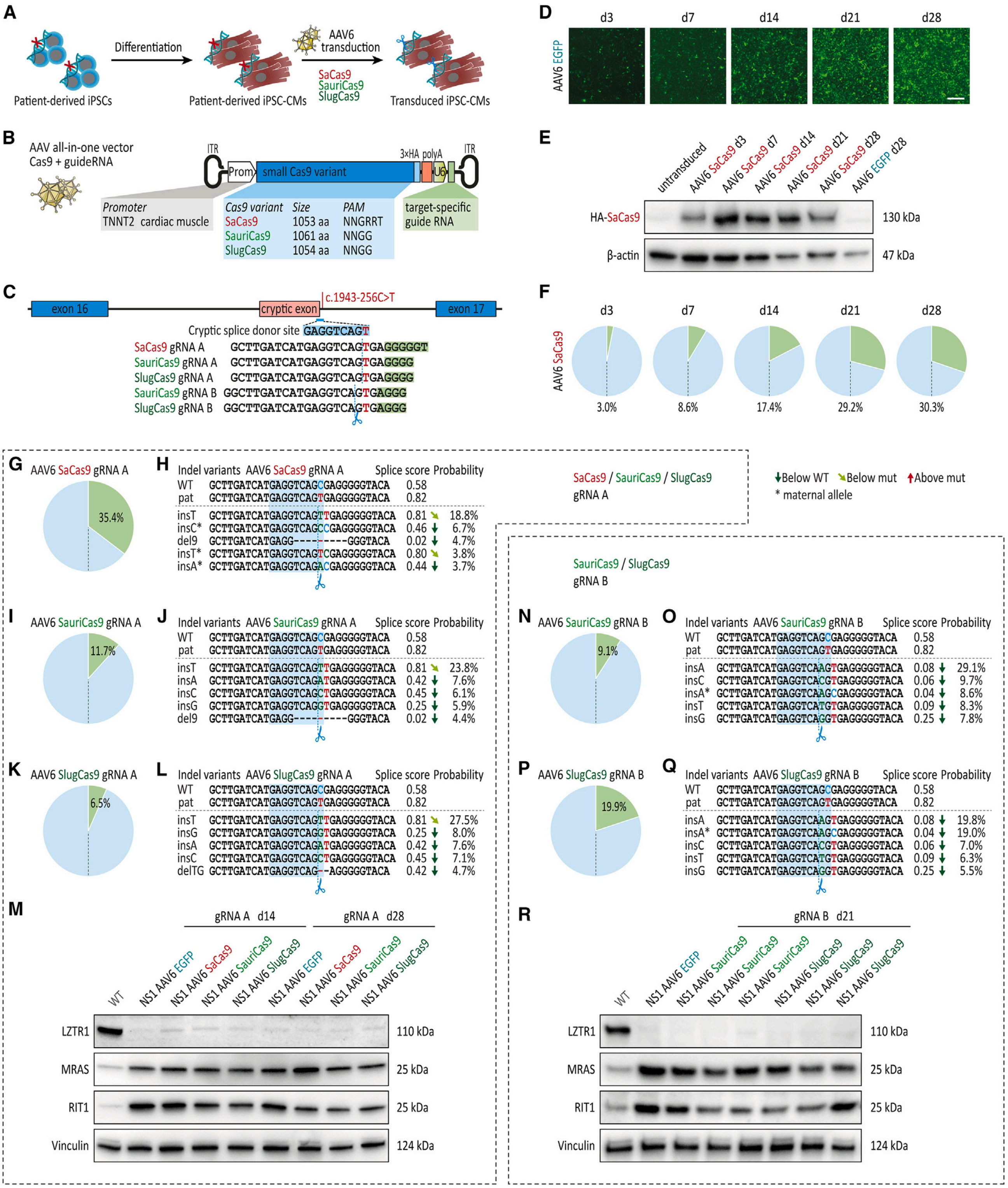


**Figure 5. All-in-one AAVs for efficient CRISPR-Cas9 editing of LZTR1 intron 16 in patient-specific iPSC-CMs**

(A) Patient-specific iPSC-CMs at day 30 of differentiation were transduced with all-in-one AAVs serotype 6 encoding small Cas9 orthologs for evaluation CRISPR-Cas9 gene editing in differentiated iPSC-CMs at different time points post-transduction; n = 2–3 independent transductions per AAV construct. (B) Schematic presentation of all-in-one

*(legend continued on next page)*

harboring distinct small Cas9 orthologs (all less than 3.3 kb in size, compared with 4.1 kb for SpCas9) under the control of a cardiac-specific troponin T promoter (*TNNT2*)[50] followed by a single guide RNA expression cassette (Figures 5A–5C). In addition to the previously used SaCas9, we included in our CRISPR screen the compact Cas9 orthologs derived from *Staphylococcus auricularis* (SauriCas9) and *Staphylococcus lugdunensis* (SlugCas9),[51,52] which recognize the simpler canonical protospacer adjacent motif *5′-NNGG-3′*, allowing for allele-specific editing with either guide RNA A or guide RNA B. To verify the all-in-one AAVs, patient-specific iPSC-CMs were transduced with AAV6-SaCas9-gRNA A or with an AAV6-GFP control vector and cultures were analyzed over time for transduction efficiency and editing efficiency (Figures 5D–5F). GFP expression increased steadily from day 3 to day 28 post-transduction with AAV6-GFP, with virtually >90% of cells showing a robust GFP signal by day 21 (Figure 5D). SaCas9 was detected in iPSC-CMs transduced with the AAV6-SaCas9-gRNA A vector at all time points measured from day 3 to day 28 (Figure 5E). Deep amplicon sequencing revealed increasing editing efficiencies over time from 3% indels at day 3 to more than 30% indels at day 28, confirming the functionality of the all-in-one AAVs (Figure 5F).

Based on these initial results, we screened the five different all-in-one AAV constructs for allele-specific editing of the mutated *LZTR1* intron 16 in patient-specific iPSC-CMs. Here, SaCas9 showed a substantially higher editing efficiency compared with SauriCas9 and SlugCas9 (Figures 5G–5L). However, as previously observed for SpCas9 with guide RNA A, all three Cas9 orthologs in combination with guide RNA A predominantly resulted in the disadvantageous insT variant after editing. Consistent with our previous data on inadequate splicing restoration as a consequence of the insT indel, no apparent LZTR1 protein expression and no reduction in RAS GTPase levels were observed in transduced iPSC-CM cultures for any of the Cas9 orthologs (Figure 5M). In contrast, editing with SauriCas9 and SlugCas9 in combination with guide RNA B in patient-specific iPSC-CMs replicated the beneficial insA as the top indel variant (Figures 5N–5Q). However, due to the low overall editing efficiency of these AAVs—at least 3 weeks after transduction—we were not able to visualize LZTR1 re-expression or a normalization of MRAS or RIT1 protein levels (Figure 5R).

Taken together, the all-in-one AAVs with the different Cas9 orthologs showed variable editing efficiencies in non-proliferative iPSC-CMs. As previously observed with the lentiviral vectors, editing in combination with guide RNA A was unable to generate therapeutically relevant indel motifs for all three small Cas9 variants. Conversely, SauriCas9 and SlugCas9 together with guide RNA B induced indel profiles that were shown to correct pathological splicing and restore LZTR1 function, thereby providing a rationale for applying these constructs as a personalized CRISPR-based gene therapy in NS patients with the pathological c.1943-256C>T variant.

## DISCUSSION

Autosomal dominant as well as autosomal recessive mutations in *LZTR1* had been recently implicated as novel causes for NS. Although all NS-associated gene mutations in components or regulators of the RAS-MAPK signaling cascade are believed to provoke signaling hyperactivity, patients harboring causative gene variants in *RAF1*, *HRAS*, *RIT1*, and *LZTR1* are particularly at risk to develop severe and early-onset HCM.[5,6] However, both pharmacological as well as invasive therapeutic options for this patient cohort are limited.[10] Off-label applications of MEK inhibitors have achieved promising results by reducing cardiac hypertrophy in first clinical case studies.[53] However, the unknown effects and potential side effects of long-term administration of these cancer drugs to infants raise serious safety concerns. Therefore, there is a need for the development of personalized targeted therapies in NS.

Although dominant *LZTR1* variants generally cluster in the Kelch motif perturbing RAS binding to the ubiquitination complex,[54] recessive *LZTR1* truncating or missense variants are distributed over the entire protein. The deep-intronic variant c.1943-256C>T discussed here appeared to occur more frequently, as several case studies reported the detection of this intronic variant combined with a second

AAVs encoding small Cas9 orthologs under control of the cardiomyocyte-specific *TNNT2* promoter together with a guide RNA expression cassette. (C) Depiction of the genome editing approach for allele-specific targeting of the deep-intronic variant in *LZTR1* intron 16 in patient-specific iPSC-CMs using different small Cas9 orthologs. (D) Representative images of patient-specific iPSC-CMs transduced with AAV6-EGFP at indicated time points post-transduction; scale bar, 500 μm. (E) Representative blots of SaCas9 levels, assessed by western blot, in patient-specific iPSC-CMs transduced with AAV6-SaCas9-guide RNA A at indicated time points post-transduction; β-actin served as loading control. (F) Analysis of editing efficiency, assessed by amplicon sequencing, in patient-specific iPSC-CMs transduced with AAV6-SaCas9-guide RNA A at indicated time points post-transduction. (G) Analysis of editing efficiency, assessed by amplicon sequencing, in patient-specific iPSC-CMs transduced with AAV6-SaCas9-guide RNA A 4 weeks post-transduction. (H) Analysis of indel variant probabilities generated by SaCas9 and guide RNA A and computational prediction of splice site motifs in AAV-transduced iPSC-CMs. (I) Analysis of editing efficiency, assessed by amplicon sequencing, in patient-specific iPSC-CMs transduced with AAV6-SauriCas9-guide RNA A 4 weeks post-transduction. (J) Analysis of indel variant probabilities generated by SauriCas9 and guide RNA A and computational prediction of splice site motifs in AAV-transduced iPSC-CMs. (K) Analysis of editing efficiency, assessed by amplicon sequencing, in patient-specific iPSC-CMs transduced with AAV6-SlugCas9-guide RNA A 4 weeks post-transduction. (L) Analysis of indel variant probabilities generated by SlugCas9 and guide RNA A and computational prediction of splice site motifs in AAV-transduced iPSC-CMs. (M) Representative blots of LZTR1, MRAS, and RIT1 levels, assessed by western blot, revealed no apparent restoration of LZTR1 function in patient-specific iPSC-CMs transduced with small Cas9 orthologs with guide RNA A 2 and 4 weeks post-transduction in comparison with iPSC-CMs transduced with AAV6-EGFP; Vinculin served as loading control. (N) Analysis of editing efficiency, assessed by amplicon sequencing, in patient-specific iPSC-CMs transduced with AAV6-SauriCas9-guide RNA B 3 weeks post-transduction. (O) Analysis of indel variant probabilities generated by SauriCas9 and guide RNA B and computational prediction of splice site motifs in AAV-transduced iPSC-CMs. (P) Analysis of editing efficiency, assessed by amplicon sequencing, in patient-specific iPSC-CMs transduced with AAV6-SlugCas9-guide RNA B 3 weeks post-transduction. (Q) Analysis of indel variant probabilities generated by SlugCas9 and guide RNA B and computational prediction of splice site motifs in AAV-transduced iPSC-CMs. (R) Representative blots of LZTR1, MRAS, and RIT1 levels, assessed by western blot, revealed no apparent restoration of LZTR1 function in patient-specific iPSC-CMs transduced with small Cas9 orthologs with guide RNA B 3 weeks post-transduction.

truncating or missense variant (such as p.Q10Afs*24, p.R688G, p.Y726*, or c.2325+G>A).[6,7] This observation was supported by gnomAD population database, since the intronic mutation was listed as the second most common NS-associated *LZTR1* variant. Considering its relatively high prevalence of 1 in 10,000 individuals in the general population, genetic therapies specifically targeting the intronic variant are of particular interest.

In this study, we utilized a preclinical disease model based on patient-specific iPSC-CMs from two NS patients to evaluate the efficacy and specificity of a CRISPR-based gene therapy targeting the intronic *LZTR1* variant c.1943-256C>T. We found that (1) both SpCas9 and SaCas9 are equally efficient and comparably selective to edit the mutated *LZTR1* intron 16 allele in proliferative iPSCs; (2) CRISPR-Cas9 editing in iPSCs corrects physiological splicing, restores LZTR1 function, and normalizes the protein levels of RAS GTPases; (3) generation of all-in-one AAVs harboring different small Cas9 orthologs allows efficient CRISPR-Cas9 genome editing in iPSC-CMs; (4) editing in non-proliferative iPSC-CMs generates distinct indel profiles compared with proliferative iPSCs; and (5) only SauriCas9 and SlugCas9 with guide RNA B reproduced therapeutically relevant indel motifs at the iPSC-CM level. Depending on the time point of CRISPR gene therapy—whether proliferative cells (e.g., stem cells in blastocysts during *in vitro* fertilization treatment) or non-proliferative cells (e.g., cardiomyocytes after postnatal diagnosis) are the primary target cell type for editing—different combinations of Cas9 and guide RNA have to be applied.

In the recent past, the clinical translation of CRISPR-Cas9-based gene therapy approaches has been initiated and it is expected that several novel therapeutics for inherited diseases will be translated clinically in the near future.[55] Two notable trials for an *in vivo* application of CRISPR-Cas9 entered clinical phase. The first aims to treat Leber congenital amaurosis—a rare condition causing early childhood blindness—by local subretinal injection of an AAV-based CRISPR-Cas9 vector targeting a cryptic exon in the *CEP290* gene (EDIT-101, NCT03872479).[14] The second example refers to a therapy for transthyretin amyloidosis, in which CRISPR-Cas9 complexes packaged in lipid nanoparticles (which mainly condense in the liver) contribute to the reduction of a misfolded protein (NTLA-2001, NCT04601051).[15] For EDIT-101, the smaller SaCas9 ortholog in combination with two guide RNAs was utilized to restore physiological splicing of the *CEP290* gene by excision of a larger intronic region flanking the cryptic exon. In contrast, for NTLA-2001 a single cut approach with SpCas9 was applied aiming to induce frameshifting indels at the CRISPR-Cas9 cleavage site in exon 2 of the transthyretin gene, thereby inducing early protein truncation and preventing further protein production.

By testing four Cas9 orthologs (SpCas9, SaCas9, SauriCas9, SlugCas9) with different guide RNA combinations, we could demonstrate that a single cut strategy was effective to genetically modify the mutated *LZTR1* intron 16. Here, both SpCas9 and SaCas9 were able to induce genetic alterations at the target site without significant differences in editing efficiency in both proliferative iPSCs and non-proliferative iPSC-CMs. In contrast to the findings of others,[51,52] SauriCa9 and SlugCas9 displayed reduced editing efficiencies compared with SaCas9. For editing at the iPSC level, SpCas9 and SaCas9 were comparably selective in predominantly binding and cleaving the mutated intron 16 allele. Despite the close similarities between the targeted and non-targeted alleles, only very low percentages of genetically altered sequences were detected on the maternal allele or in transfected WT cells, demonstrating the high degree of specificity when the CRISPR-Cas9 components were delivered as a ribonucleoprotein complex. On the contrary, editing of the maternal non-target allele was significantly increased in patient-specific iPSC-CMs for all small Cas9 orthologs (up to 19% for SlugCas9 with guide RNA B), most likely due to the constant long-term exposure of the cultures to the Cas9 system by the AAVs. However, since in clinical cases with biallelic *LZTR1* variants, the second allele is generally non-functional (in our case by carrying a truncating variant in exon 1), its potential editing would have no functional consequences. In addition to the low off-target activity on the non-targeted allele, we observed no off-target editing on the 10 most likely off-target sites outside of *LZTR1* intron 16 for both guide RNAs after editing with SpCas9 or SaCas9.

Importantly for clinical translation, the indel profiles and indel frequencies upon CRISPR-Cas9 editing were robustly reproducible in both patient-specific iPSCs and iPSC-CMs. Of note, we observed profound differences between the indel profiles generated by guide RNA A and guide RNA B (regardless of the Cas9 ortholog used), even though their cut sites were separated by just 1 base pair, suggesting that the exact Cas9 cleavage site is the major determinant factor over the indel profile rather than the Cas9 enzyme itself.[56] Although the indel profiles were dominated by a highly frequent top indel variant in both cell types, the top indel variants were not consistently identical between iPSCs and iPSC-CMs. While editing with SpCas9, SauriCas9, and SlugCas9 in combination with guide RNA B resulted in the identical top indel variant (insA) in both iPSCs and iPSC-CMs, editing with all four Cas9 orthologs in combination with guide RNA A generated critically distinct top indel variants in patient-specific iPSCs (del9) vs. iPSC-CMs (insT), indicating the activity of different DNA repair mechanisms in proliferative and non-proliferative cells, respectively. While top indel insT in iPSC-CMs was a consequence of cNHEJ (active in both proliferative and non-proliferative cells), del9 in iPSCs most likely resulted from MMEJ, a DNA double-strand break repair mechanism known to be predominantly active in proliferating cells during S and G2 phase.[57,58]

Using monoclonal iPSC-CMs harboring the top indel variants, correction of pathological splicing, restoration of LZTR1 function, and normalization of RAS protein accumulation to WT levels were observed for the del9 and insA indel variants. Importantly, and in line with previous reports by our group and others,[7,44,46] one functional *LZTR1* allele was sufficient to regulate the pool of RAS GTPases. In contrast, analysis of the indel variant insT (the top variant induced by guide RNA A in iPSC-CMs) failed to disrupt the cryptic donor splice site and to rescue LZTR1 function, highlighting the significance of cell type-specific screens for CRISPR-based gene therapies. Using lentiviral vectors and all-in-one AAVs,

robust editing of the intronic *LZTR1* locus was achieved in transduced iPSC-CMs cultures. Although editing in combination with guide RNA B generated the same beneficial top indel variant (insA) in iPSC-CMs, editing efficiencies in virally transduced iPSC-CMs may be too low to detect efficient restoration of LZTR1 proteins. Thus, higher editing efficiencies are needed to effectively rehabilitate the core pathological mechanism of a hyperactivated RAS-MAPK signaling pathway in cardiomyocytes.

In summary, by utilizing a patient-specific iPSC-CM screening platform to test CRISPR-based therapeutic approaches targeting a deep-intronic *LZTR1* variant c.1943-256C>T, this study demonstrated crucial differences between CRISPR-Cas9 gene editing in proliferative iPSCs vs. non-proliferative iPSC-CMs. Editing outcomes appeared to depend not only on the particular Cas9 cleavage site, but also on the target cell type. Although editing was highly reproducible for each approach, the most frequent indel variants were not necessarily identical across cell types, highlighting the importance of cell type-specific screens for preclinical testing of CRISPR therapeutics.

## MATERIALS AND METHODS

### Ethical approval

The study was approved by the Ethics Committee of the University Medical Center Göttingen (approval number: 10/9/15) and carried out in accordance with the approved guidelines. Written informed consent was obtained from all participants or their legal representatives prior to the participation in the study.

### Generation and culture of human iPSCs

Human iPSC lines from a healthy donor and from two NS patients with pathological biallelic truncating variants in *LZTR1*, as well as CRISPR-Cas9-edited iPSC lines were used in this study. WT iPSC line UMGi014-C clone 14 (isWT1.14, here abbreviated as WT) was generated from dermal fibroblasts from a male donor using the integration-free Sendai virus and was described previously.[59] Patient-specific iPSC lines UMGi030-A clone 14 (isHOCMx1.14, here abbreviated as NS1), UMGi031-A clone 8 (isHOCMx2.8, here abbreviated as NS2), UMGi032-A clone 17 (isHOCMx-R1.17, here abbreviated as NS-R1), and UMGi033-A clone 9 (isHOCMx-R2.9, here abbreviated as NS-R2) were generated from patient dermal fibroblasts using the integration-free Sendai virus according to the manufacturer's instructions with modifications and were described previously.[7] Human iPSCs were cultured in feeder-free and serum-free culture conditions in StemMACS iPS-Brew XF medium (Miltenyi Biotec) or StemFlex medium (Thermo Fisher Scientific) on Matrigel-coated (growth factor reduced, BD Biosciences) plates in a humidified incubator at 37°C and 5% $CO_2$.

### CRISPR-Cas9 genome editing of human iPSCs

Therapeutic genome editing in patient-specific iPSCs was performed by targeting the paternal variant c.1943-256C>T in intron 16 of the *LZTR1* gene using ribonucleoprotein (RNP)-based CRISPR-Cas9. The iPSCs were cultured in StemFlex medium on Matrigel-coated plates and transfected between passage 12 and 20. The CRISPR-Cas9 RNP complex was assembled either by mixing of the individual Alt-R CRISPR-Cas9 crRNA and the Alt-R CRISPR-Cas9 tracrRNA (preassembled in a 1:1 ratio) with the Alt-R SpCas9 Nuclease 3NLS (IDT DNA Technologies) or by mixing of the individual single guide RNA (sgRNA) with Alt-R SpCas9 nuclease (IDT DNA Technologies) or EnGen SaCas9 Nuclease (New England Biolabs) at 1:3 M ratio, incubated for 10 min at room temperature (RT) and diluted in nucleofector solution. Twenty minutes before nucleofection, iPSCs at a confluence of 70%–80% were pretreated with 2 μM Thiazovivin (Merck Millipore) and dissociated using Versene solution (Thermo Fisher Scientific). Nucleofection was performed with $2 \times 10^6$ iPSCs using the 4D Amaxa Nucleofector system (Lonza; program CA-137) and the P3 Primary Cell 4D-Nucleofector X Kit (Lonza) according to manufacturer's instructions. Following nucleofection, iPSCs were replated into a Matrigel-coated well of a six-well plate containing StemFlex medium supplemented with 2 μM Thiazovivin and 100 U/mL penicillin and 100 μg/mL streptomycin (Thermo Fisher Scientific). After 3–4 days, genome editing events and efficiencies of transfected bulks were analyzed by Sanger sequencing or amplicon sequencing. Guide RNA target sequences are listed in Table S1.

To establish clonal iPSC lines with specific indel variants, transfected iPSCs were singularized using the single cell dispenser CellenOne (Cellenion/Scienion) in StemFlex medium on Matrigel-coated 96-well plates. Successful genome editing was identified by Sanger sequencing and CRISPR-edited isogenic iPSC lines UMGi030-A-1 clone 34 (isHOCMx1-corr.34, here abbreviated as NS1-del9), UMGi030-A-2 clone 12 (isHOCMx1-corr2.12, here abbreviated as NS1-insA), UMGi030-A-3 clone 2 and clone 3 (isHOCMx1-LZTR1-In16-Tins.2/3, here abbreviated as NS1-insT), UMGi031-A-1 clone 17 (isHOCMx2-corr.17, here abbreviated as NS2-del9), UMGi031-A-2 clone 13 (isHOCMx2-corr2.13, here abbreviated as NS2-insA) were expanded and maintained in StemMACS iPS-Brew XF medium on Matrigel-coated plates for at least 10 passages prior to molecular karyotyping, pluripotency characterization, and differentiation experiments.

### Pluripotency characterization of human iPSCs

Pluripotency analysis was performed via immunocytochemistry and flow cytometry, as previously described with modifications.[7] For molecular karyotyping, genomic DNA of iPSC clones was sent for genome-wide analysis via Illumina BeadArray (Life&Brain, Germany). Digital karyotypes were analyzed in GenomeStudio v2.0 software (Illumina). For off-target screening, the top 10 predicted off-target regions for the respective guide RNA ranked by the CFD off-target score using CRISPOR[60] were analyzed by Sanger sequencing. Primer sequences are listed in Table S2. Antibodies used for immunofluorescence and flow cytometry are listed in Table S3.

### Cardiomyocyte differentiation of iPSCs

Human iPSC lines were differentiated into ventricular iPSC-CMs via WNT signaling modulation and subsequent metabolic selection, as previously described,[47] and cultivated in feeder-free and serum-free

culture conditions at least until day 30 post-differentiation before being used for molecular experiments.

### Lentiviral transduction of iPSC-CMs

LentiCRISPRv2GFP was a gift from David Feldser (Addgene plasmid #82416; RRID:Addgene_82416), Lenti_SaCRISPR_GFP was a gift from Christopher Vakoc (Addgene plasmid #118636; RRID:Addgene_118636), pMD2.G was a gift from Didier Trono (Addgene plasmid #12259; RRID:Addgene_12259), and psPAX2 was a gift from Didier Trono (Addgene plasmid #12260; RRID:Addgene_12260). Guide RNA oligonucleotides were cloned into lentiviral plasmids using BsmBI (Thermo Fisher Scientific). Guide RNA sequences are listed in Table S1 and plasmids are listed in Table S4. Lentiviral particles were produced in HEK293T cells transfected with transfer and helper plasmids using Lipofectamine 3000 (Thermo Fisher Scientific) according to the manufacturer's instructions. Virus was harvested from day 2 to day 5 post-transfection by medium collection and centrifugation at 500 × *g* at 4°C for 5 min. Collected virus was filtered using a 0.45-μm filter and a syringe. Lentiviral transduction of iPSC-CM cultures was performed in culture medium supplemented with 100 U/mL penicillin, 100 μg/mL streptomycin (Thermo Fisher Scientific), and 10 μg/mL Polybrene Transfection Reagent (Merck) and cells were transduced with 1.5 mL of the respective lentivirus. After 24 h of incubation, medium was replaced with cardio culture medium and cells were maintained for an additional 4 weeks post-infection.

### AAV transduction of iPSC-CMs

For generation of all-in-one AAV plasmids, the CMV promoter of pAAV-CMV-SauriCas9 (a gift from Yongming Wang; Addgene plasmid #135964; RRID: Addgene_135964) was exchanged by the cardiomyocyte-specific *TNNT2* promoter region[50] using XbaI and BshTI (Thermo Fisher Scientific). The SaCas9 and SlugCas9 open reading frames were amplified from pX601-miniCMV-SaCas9-U6-LacZvsSaCas9 (a gift from Alex Hewitt; Addgene plasmid #107049; RRID: Addgene_107049) and pAAV-CMV-SlugCas9 (a gift from Yongming Wang; Addgene plasmid #163796; RRID: Addgene_163796) by infusion cloning (Takara) and BshTI and BamHI (Thermo Fisher Scientific) in pAAV-TNNT2-SauriCas9. Guide RNA oligonucleotides were cloned into AAV plasmids using Eco31I (Thermo Fisher Scientific). ITRs were verified by XmaI and KpnI (New England Biolabs) restriction digests prior to AAV production. Guide RNA sequences are listed in Table S1 and plasmids are listed in Table S4. Recombinant AAV particles of serotype 6 (AAV-6) were produced in transiently transfected HEK293T cells and were purified from the cell lysate by iodixanol step gradient ultracentrifugation followed by heparin affinity chromatography on an Äkta FPLC. The peak eluate was dialyzed against PBS overnight and frozen in single-use aliquots at −80°C. Purity of viral particles was confirmed to be >98% by SDS-PAGE, and vector genome titer was assessed by qPCR. AAV transduction of iPSC-CM cultures was performed in culture medium supplemented with 100 U/mL penicillin and 100 μg/mL streptomycin (Thermo Fisher Scientific) and cells were transduced at a multiplicity of infection of $1 \times 10^5$. After 24 h of incubation, medium was replaced with cardio culture medium and cells were maintained for up to 4 weeks post-infection.

### Amplicon sequencing of genome-edited iPSCs and iPSC-CMs

Amplicon sequencing was performed on genomic DNA of iPSCs acquired 3–4 days post-transfection with the CRISPR-Cas9 complexes and at various indicated time points after lentiviral or AAV transduction of iPSC-CMs. Diluted genomic DNA was used as PCR template and *LZTR1* intron 16 was amplified using the GoTaq G2 DNA polymerase (Promega) according to the manufacturer's instructions and amplicons were subjected to Illumina-based amplicon sequencing (Genewiz/Azenta Life Sciences). Obtained sequences were aligned to the patients' target allele including 40 base pairs upstream and 40 base pairs downstream flanking the disease-causing variant c.1943-256C>T by using the online tool Cas-analyzer.[61] Filtered sequences containing both indicator sequences (at least 10,000 paired-end reads per sample) were manually categorized according to their allele of origin (target vs. non-target allele) and type of introduced mutation (WT, base pair exchange, deletion, insertion or deletion plus insertion). Genetic changes (indels and base pair exchange) within an editing window of 5 base pairs up- or downstream of the cleavage site were considered a result of CRISPR-Cas9-induced editing, whereas base pair exchanges detected outside of the editing window were considered sequencing artifacts. For computational predictions of indel events, the web-based tool inDelphi[28] was utilized. For computational prediction of splice site motifs, the SpliceAI online tool[45,62] was utilized.

### Immunocytochemistry of iPSC-CMs

For immunofluorescence studies, iPSC-CMs cultured on glass coverslips were fixed in Roti-Histofix 4% (Carl Roth) at RT for 10 min and blocked with 1% bovine serum albumin (BSA; Sigma-Aldrich) in PBS (Thermo Fisher Scientific) at 4°C overnight. Primary antibodies were applied in 1% BSA in PBS at 4°C overnight. Secondary antibodies with minimal cross reactivity were administered in 1% BSA in PBS at RT for 1 h. Cells were permeabilized with 0.1% Triton X-100 (Carl Roth) in staining solution. Nuclei were counter-stained with 8.1 μM Hoechst 33342 (Thermo Fisher Scientific) at RT for 10 min. Samples were mounted in Fluoromount-G (Thermo Fisher Scientific). Images were collected using the Axio Imager M2 microscopy system (Carl Zeiss) and Zen 2.3 software. Antibodies used for immunocytochemistry are listed in Table S3.

### Western blot analysis of iPSC-CMs

For western blot analysis, iPSC-CMs were pelleted by scratching at indicated time points of differentiation and collected in RIPA buffer (Thermo Fisher Scientific) containing phosphatase and protease inhibitor (Thermo Fisher Scientific) and snap-frozen in liquid nitrogen. Protein containing supernatant was collected by centrifugation at 15,000 × *g* for 10 min. Protein concentration was determined by BCA assay (Thermo Fisher Scientific) according to the manufacturer's instructions. Samples were denatured at 95°C for 5 min. Fifteen micrograms of protein was loaded onto a 4%–15% Mini-PROTEAN TGX Stain-Free precast gel (Bio-Rad). Protein was separated by SDS-PAGE by applying 200 V for 30 min. Post-running, TGX gels were activated via UV light application using the Trans-Blot Turbo transfer system (Bio-Rad). While blotting, proteins were

transferred to a nitrocellulose membrane (25 V constant, 1.3 A for 7 min). Total protein amount was detected via the ChemiDoc XRS+ (Bio-Rad) system and used for protein normalization. After 1 h in blocking solution (5% milk in TBS-T, Sigma-Aldrich), membranes were incubated in primary antibody solution (1% milk in TBS-T) overnight. Membrane was washed thrice with TBS-T before applying the secondary antibody (1:10,000 in 1% milk in TBS-T) at RT for 1 h. After washing, signals were detected upon application of SuperSignal West Femto Maximum Sensitivity Substrate (Thermo Fisher Scientific). Image acquisition was performed with the ChemiDoc XRS+ (Bio-Rad) using the high-resolution mode. For protein quantification, ImageLab (Bio-Rad) was used and protein levels were normalized to total protein and second to the corresponding WT samples on each blot. For each iPSC line, four individual differentiations were analyzed, and corresponding samples were pooled for quantitative analysis. All antibodies used for western blot are listed in Table S3.

### Reverse-transcriptase PCR and real-time PCR analysis of iPSC-CMs

Pellets of iPSC-CMs collected at day 30 of differentiation were snap-frozen in liquid nitrogen and stored at −80°C. Total RNA was isolated using the NucleoSpin RNA Mini kit (Macherey-Nagel) according to the manufacturer's instructions; 200 ng RNA was used for the first-strand cDNA synthesis by using the MULV Reverse Transcriptase and Oligo d(T)16 (Thermo Fisher Scientific). For reverse-transcriptase PCR, one-tenth of cDNA was used as PCR template and amplified using the GoTaq G2 DNA polymerase (Promega) according to the manufacturer's instructions. For quantification of splicing, the ratio between signal intensity of the cryptic exon and the non-cryptic variant of the corresponding sample was calculated using ImageLab (Bio-Rad). For real-time PCR, cDNA was diluted 1:1 with nuclease-free water (Promega). Quantitative real-time PCR reactions were carried out using the SYBR Green PCR master mix and ROX Passive Reference Dye (Bio-Rad) with Micro-Amp Optical 384-well plates, and the 7900HT fast real-time PCR system (Applied Biosystems) according to the manufacturer's instructions with the following parameters: 95°C for 10 min, followed by 40 cycles at 95°C for 15 s and 60°C for 1 min. Analysis was conducted using the ΔΔCT method and values were normalized to *GAPDH, TUBB5*, and *RPL37A* gene expression and to WT controls. For each iPSC line, three individual differentiations were analyzed, and corresponding samples were pooled for quantitative analysis. For evaluation of nonsense-mediated decay, the expression of the maternal and the paternal transcript (distinguished by the mutation in exon 1) was analyzed using amplicon sequencing. Primer sequences are listed in Table S2.

### Statistics

Data are presented as the mean ± standard error of the mean. Statistical comparisons were performed using the D'Agostino-Pearson normality test and the nonparametric Kruskal-Wallis test followed by Dunn's multiple comparisons test in Prism 10 (GraphPad). Results were considered statistically significant when the p value was $\leq 0.05$.

## DATA AND CODE AVAILABILITY

All human iPSC lines used in this study are deposited in the stem cell biobank of the University Medical Center Göttingen and are available for research use upon request. Sequencing data are available upon request.

## SUPPLEMENTAL INFORMATION

Supplemental information can be found online at https://doi.org/10.1016/j.omtn.2024.102123.

## ACKNOWLEDGMENTS

We thank Laura Cyganek, Yvonne Hintz, Nadine Gotzmann, Olga Kowatsch, Lisa Krebs, and Yvonne Wedekind (Stem Cell Unit, University Medical Center Göttingen) for excellent technical assistance. This work was supported by the Else Kröner-Fresenius Foundation: project number 2019_A75 to L.C., by the German Research Foundation (DFG): project number 417880571 to L.C.; project number 501985000 to L.C.; project number 193793266, Collaborative Research Centre 1002, C04, C07, D01, D02 and S01 to W.H.Z., L.C.Z., G.H., B.W., and L.C.; project number 390729940, Germany's Excellence Strategy - EXC 2067/1 to G.H., W.H.Z., B.W., and L.C.; by the German Federal Ministry of Education and Research (BMBF)/German Center for Cardiovascular Research (DZHK): project numbers 81X2300171, 81X2300191, 81X3300113, 81Z0300112, and 81Z030116 to H.H., L.R., E.S., G.H., W.H.Z., B.W., and L.C.; by the Foundation Leducq (20CVD04) to W.H.Z.; by the German Heart Foundation (Deutsche Herzstiftung) to L.R.; and by the German Cardiac Society (DGK) to H.H.

## AUTHOR CONTRIBUTIONS

L.C. designed the study and the experiments. C.K., H.H., E.S., S.K., L.R., and L.C. performed the experiments and analyzed the data. L.C.Z., G.H., W.H.Z., and B.W. gave technical support and conceptual advice. L.C., C.K., and H.H. wrote and edited the manuscript.

## DECLARATION OF INTERESTS

The authors declare no competing interests.

## REFERENCES


1. Roberts, A.E., Allanson, J.E., Tartaglia, M., and Gelb, B.D. (2013). Noonan syndrome. Lancet *381*, 333–342. https://doi.org/10.1016/S0140-6736(12)61023-X.
2. Linglart, L., and Gelb, B.D. (2020). Congenital heart defects in Noonan syndrome: Diagnosis, management, and treatment. Am. J. Med. Genet. C Semin. Med. Genet. *184*, 73–80. https://doi.org/10.1002/ajmg.c.31765.
3. Hickey, E.J., Mehta, R., Elmi, M., Asoh, K., McCrindle, B.W., Williams, W.G., Manlhiot, C., and Benson, L. (2011). Survival implications: hypertrophic cardiomyopathy in Noonan syndrome: Hypertrophic cardiomyopathy in Noonan syndrome. Congenit. Heart Dis. *6*, 41–47. https://doi.org/10.1111/j.1747-0803.2010.00465.x.
4. Wilkinson, J.D., Lowe, A.M., Salbert, B.A., Sleeper, L.A., Colan, S.D., Cox, G.F., Towbin, J.A., Connuck, D.M., Messere, J.E., and Lipshultz, S.E. (2012). Outcomes in children with Noonan syndrome and hypertrophic cardiomyopathy: a study from the Pediatric Cardiomyopathy Registry: A study from the Pediatric Cardiomyopathy Registry. Am. Heart J. *164*, 442–448. https://doi.org/10.1016/j.ahj.2012.04.018.
5. Leoni, C., Blandino, R., Delogu, A.B., De Rosa, G., Onesimo, R., Verusio, V., Marino, M.V., Lanza, G.A., Rigante, D., Tartaglia, M., and Zampino, G. (2022).

Genotype-cardiac phenotype correlations in a large single-center cohort of patients affected by RASopathies: Clinical implications and literature review. Am. J. Med. Genet. *188*, 431–445. https://doi.org/10.1002/ajmg.a.62529.

6. Johnston, J.J., van der Smagt, J.J., Rosenfeld, J.A., Pagnamenta, A.T., Alswaid, A., Baker, E.H., Blair, E., Borck, G., Brinkmann, J., Craigen, W., et al. (2018). Autosomal recessive Noonan syndrome associated with biallelic LZTR1 variants. Genet. Med. *20*, 1175–1185. https://doi.org/10.1038/gim.2017.249.
7. Hanses, U., Kleinsorge, M., Roos, L., Yigit, G., Li, Y., Barbarics, B., El-Battrawy, I., Lan, H., Tiburcy, M., Hindmarsh, R., et al. (2020). Intronic CRISPR Repair in a Preclinical Model of Noonan Syndrome-Associated Cardiomyopathy. Circulation *142*, 1059–1076. https://doi.org/10.1161/CIRCULATIONAHA.119.044794.
8. Bigenzahn, J.W., Collu, G.M., Kartnig, F., Pieraks, M., Vladimer, G.I., Heinz, L.X., Sedlyarov, V., Schischlik, F., Fauster, A., Rebsamen, M., et al. (2018). LZTR1 is a regulator of RAS ubiquitination and signaling. Science *362*, 1171–1177. https://doi.org/10.1126/science.aap8210.
9. Castel, P., Cheng, A., Cuevas-Navarro, A., Everman, D.B., Papageorge, A.G., Simanshu, D.K., Tankka, A., Galeas, J., Urisman, A., and McCormick, F. (2019). RIT1 oncoproteins escape LZTR1-mediated proteolysis. Science *363*, 1226–1230. https://doi.org/10.1126/science.aav1444.
10. Ommen, S.R., Mital, S., Burke, M.A., Day, S.M., Deswal, A., Elliott, P., Evanovich, L.L., Hung, J., Joglar, J.A., Kantor, P., et al. (2020). 2020 AHA/ACC Guideline for the Diagnosis and Treatment of Patients With Hypertrophic Cardiomyopathy: A Report of the American College of Cardiology/American Heart Association Joint Committee on Clinical Practice Guidelines. J. Am. Coll. Cardiol. *76*, e159–e240. https://doi.org/10.1016/j.jacc.2020.08.045.
11. Komor, A.C., Badran, A.H., and Liu, D.R. (2017). CRISPR-Based Technologies for the Manipulation of Eukaryotic Genomes. Cell *168*, 20–36. https://doi.org/10.1016/j.cell.2016.10.044.
12. van Kampen, S.J., and van Rooij, E. (2019). CRISPR Craze to Transform Cardiac Biology. Trends Mol. Med. *25*, 791–802. https://doi.org/10.1016/j.molmed.2019.06.008.
13. Frangoul, H., Altshuler, D., Cappellini, M.D., Chen, Y.-S., Domm, J., Eustace, B.K., Foell, J., de la Fuente, J., Grupp, S., Handgretinger, R., et al. (2021). CRISPR-Cas9 Gene Editing for Sickle Cell Disease and β-Thalassemia. N. Engl. J. Med. *384*, 252–260. https://doi.org/10.1056/NEJMoa2031054.
14. Maeder, M.L., Stefanidakis, M., Wilson, C.J., Baral, R., Barrera, L.A., Bounoutas, G.S., Bumcrot, D., Chao, H., Ciulla, D.M., DaSilva, J.A., et al. (2019). Development of a gene-editing approach to restore vision loss in Leber congenital amaurosis type 10. Nat. Med. *25*, 229–233. https://doi.org/10.1038/s41591-018-0327-9.
15. Gillmore, J.D., Gane, E., Taubel, J., Kao, J., Fontana, M., Maitland, M.L., Seitzer, J., O'Connell, D., Walsh, K.R., Wood, K., et al. (2021). CRISPR-Cas9 In Vivo Gene Editing for Transthyretin Amyloidosis. N. Engl. J. Med. *385*, 493–502. https://doi.org/10.1056/NEJMoa2107454.
16. Long, C., Li, H., Tiburcy, M., Rodriguez-Caycedo, C., Kyrychenko, V., Zhou, H., Zhang, Y., Min, Y.-L., Shelton, J.M., Mammen, P.P.A., et al. (2018). Correction of diverse muscular dystrophy mutations in human engineered heart muscle by single-site genome editing. Sci. Adv. *4*, eaap9004. https://doi.org/10.1126/sciadv.aap9004.
17. Amoasii, L., Hildyard, J.C.W., Li, H., Sanchez-Ortiz, E., Mireault, A., Caballero, D., Harron, R., Stathopoulou, T.-R., Massey, C., Shelton, J.M., et al. (2018). Gene editing restores dystrophin expression in a canine model of Duchenne muscular dystrophy. Science *362*, 86–91. https://doi.org/10.1126/science.aau1549.
18. Moretti, A., Fonteyne, L., Giesert, F., Hoppmann, P., Meier, A.B., Bozoglu, T., Baehr, A., Schneider, C.M., Sinnecker, D., Klett, K., et al. (2020). Somatic gene editing ameliorates skeletal and cardiac muscle failure in pig and human models of Duchenne muscular dystrophy. Nat. Med. *26*, 207–214. https://doi.org/10.1038/s41591-019-0738-2.
19. Beyret, E., Liao, H.-K., Yamamoto, M., Hernandez-Benitez, R., Fu, Y., Erikson, G., Reddy, P., and Izpisua Belmonte, J.C. (2019). Single-dose CRISPR-Cas9 therapy extends lifespan of mice with Hutchinson-Gilford progeria syndrome. Nat. Med. *25*, 419–422. https://doi.org/10.1038/s41591-019-0343-4.
20. Santiago-Fernández, O., Osorio, F.G., Quesada, V., Rodríguez, F., Basso, S., Maeso, D., Rolas, L., Barkaway, A., Nourshargh, S., Folgueras, A.R., et al. (2019). Development of a CRISPR/Cas9-based therapy for Hutchinson-Gilford progeria syndrome. Nat. Med. *25*, 423–426. https://doi.org/10.1038/s41591-018-0338-6.
21. Fu, Y., Foden, J.A., Khayter, C., Maeder, M.L., Reyon, D., Joung, J.K., and Sander, J.D. (2013). High-frequency off-target mutagenesis induced by CRISPR-Cas nucleases in human cells. Nat. Biotechnol. *31*, 822–826. https://doi.org/10.1038/nbt.2623.
22. Zuccaro, M.V., Xu, J., Mitchell, C., Marin, D., Zimmerman, R., Rana, B., Weinstein, E., King, R.T., Palmerola, K.L., Smith, M.E., et al. (2020). Allele-Specific Chromosome Removal after Cas9 Cleavage in Human Embryos. Cell *183*, 1650–1664.e15. https://doi.org/10.1016/j.cell.2020.10.025.
23. Cradick, T.J., Fine, E.J., Antico, C.J., and Bao, G. (2013). CRISPR/Cas9 systems targeting β-globin and CCR5 genes have substantial off-target activity. Nucleic Acids Res. *41*, 9584–9592. https://doi.org/10.1093/nar/gkt714.
24. Bétermier, M., Bertrand, P., and Lopez, B.S. (2014). Is non-homologous end-joining really an inherently error-prone process? PLoS Genet. *10*, e1004086. https://doi.org/10.1371/journal.pgen.1004086.
25. Johansen, A.K., Molenaar, B., Versteeg, D., Leitoguinho, A.R., Demkes, C., Spanjaard, B., de Ruiter, H., Akbari Moqadam, F., Kooijman, L., Zentilin, L., et al. (2017). Postnatal Cardiac Gene Editing Using CRISPR/Cas9 With AAV9-Mediated Delivery of Short Guide RNAs Results in Mosaic Gene Disruption. Circ. Res. *121*, 1168–1181. https://doi.org/10.1161/CIRCRESAHA.116.310370.
26. Chakrabarti, A.M., Henser-Brownhill, T., Monserrat, J., Poetsch, A.R., Luscombe, N.M., and Scaffidi, P. (2019). Target-Specific Precision of CRISPR-Mediated Genome Editing. Mol. Cell *73*, 699–713.e6. https://doi.org/10.1016/j.molcel.2018.11.031.
27. van Overbeek, M., Capurso, D., Carter, M.M., Thompson, M.S., Frias, E., Russ, C., Reece-Hoyes, J.S., Nye, C., Gradia, S., Vidal, B., et al. (2016). DNA Repair Profiling Reveals Nonrandom Outcomes at Cas9-Mediated Breaks. Mol. Cell *63*, 633–646. https://doi.org/10.1016/j.molcel.2016.06.037.
28. Shen, M.W., Arbab, M., Hsu, J.Y., Worstell, D., Culbertson, S.J., Krabbe, O., Cassa, C.A., Liu, D.R., Gifford, D.K., and Sherwood, R.I. (2018). Predictable and precise template-free CRISPR editing of pathogenic variants. Nature *563*, 646–651. https://doi.org/10.1038/s41586-018-0686-x.
29. Allen, F., Crepaldi, L., Alsinet, C., Strong, A.J., Kleshchevnikov, V., De Angeli, P., Páleníková, P., Khodak, A., Kiselev, V., Kosicki, M., et al. (2018). Predicting the mutations generated by repair of Cas9-induced double-strand breaks. Nat. Biotechnol. *37*, 64–72. https://doi.org/10.1038/nbt.4317.
30. Cornu, T.I., Mussolino, C., and Cathomen, T. (2017). Refining strategies to translate genome editing to the clinic. Nat. Med. *23*, 415–423. https://doi.org/10.1038/nm.4313.
31. Sayed, N., Liu, C., and Wu, J.C. (2016). Translation of Human-Induced Pluripotent Stem Cells: From Clinical Trial in a Dish to Precision Medicine: From Clinical Trial in a Dish to Precision Medicine. J. Am. Coll. Cardiol. *67*, 2161–2176. https://doi.org/10.1016/j.jacc.2016.01.083.
32. van Mil, A., Balk, G.M., Neef, K., Buikema, J.W., Asselbergs, F.W., Wu, S.M., Doevendans, P.A., and Sluijter, J.P.G. (2018). Modelling inherited cardiac disease using human induced pluripotent stem cell-derived cardiomyocytes: progress, pitfalls, and potential. Cardiovasc. Res. *114*, 1828–1842. https://doi.org/10.1093/cvr/cvy208.
33. Fomin, A., Gärtner, A., Cyganek, L., Tiburcy, M., Tuleta, I., Wellers, L., Folsche, L., Hobbach, A.J., von Frieling-Salewsky, M., Unger, A., et al. (2021). Truncated titin proteins and titin haploinsufficiency are targets for functional recovery in human cardiomyopathy due to TTN mutations. Sci. Transl. Med. *13*, eabd3079. https://doi.org/10.1126/scitranslmed.abd3079.
34. Pagnamenta, A.T., Kaisaki, P.J., Bennett, F., Burkitt-Wright, E., Martin, H.C., Ferla, M.P., Taylor, J.M., Gompertz, L., Lahiri, N., Tatton-Brown, K., et al. (2019). Delineation of dominant and recessive forms of LZTR1-associated Noonan syndrome. Clin. Genet. *95*, 693–703. https://doi.org/10.1111/cge.13533.
35. Perin, F., Trujillo-Quintero, J.P., Jimenez-Jaimez, J., Rodríguez-Vázquez Del Rey, M.D.M., Monserrat, L., and Tercedor, L. (2019). Two Novel Cases of Autosomal Recessive Noonan Syndrome Associated With LZTR1 Variants. Rev. Esp. Cardiol. *72*, 978–980. https://doi.org/10.1016/j.rec.2019.05.002.
36. Güemes, M., Martín-Rivada, Á., Ortiz-Cabrera, N.V., Martos-Moreno, G.Á., Pozo-Román, J., and Argente, J. (2019). LZTR1: Genotype Expansion in Noonan Syndrome. Horm. Res. Paediatr. *92*, 269–275.

37. Ferrari, L., Mangano, E., Bonati, M.T., Monterosso, I., Capitanio, D., Chiappori, F., Brambilla, I., Gelfi, C., Battaglia, C., Bordoni, R., and Riva, P. (2020). Digenic inheritance of subclinical variants in Noonan Syndrome patients: an alternative pathogenic model? Eur. J. Hum. Genet. *28*, 1432–1445. https://doi.org/10.1038/s41431-020-0658-0.

38. Yamamoto, G.L., Aguena, M., Gos, M., Hung, C., Pilch, J., Fahiminiya, S., Abramowicz, A., Cristian, I., Buscarilli, M., Naslavsky, M.S., et al. (2015). Rare variants in SOS2 and LZTR1 are associated with Noonan syndrome. J. Med. Genet. *52*, 413–421. https://doi.org/10.1136/jmedgenet-2015-103018.

39. Jacquinet, A., Bonnard, A., Capri, Y., Martin, D., Sadzot, B., Bianchi, E., Servais, L., Sacré, J.P., Cavé, H., and Verloes, A. (2020). Oligo-astrocytoma in LZTR1-related Noonan syndrome. Eur. J. Med. Genet. *63*, 103617. https://doi.org/10.1016/j.ejmg.2019.01.007.

40. Umeki, I., Niihori, T., Abe, T., Kanno, S.-I., Okamoto, N., Mizuno, S., Kurosawa, K., Nagasaki, K., Yoshida, M., Ohashi, H., et al. (2019). Delineation of LZTR1 mutation-positive patients with Noonan syndrome and identification of LZTR1 binding to RAF1-PPP1CB complexes. Hum. Genet. *138*, 21–35. https://doi.org/10.1007/s00439-018-1951-7.

41. Ghedira, N., Kraoua, L., Lagarde, A., Abdelaziz, R.B., Olschwang, S., Desvignes, J.P., Abdelhak, S., Monastiri, K., Levy, N., SandreGiovannoli, A.D., and Mrad, R. (2017). Further Evidence for the Implication of LZTR1, a Gene not Associated with the Ras-Mapk Pathway, in the Pathogenesis of Noonan Syndrome. Biol. Med. *9*, 4–7. https://doi.org/10.4172/0974-8369.1000414.

42. Chen, H., Li, X., Liu, X., Wang, J., Zhang, Z., Wu, J., Huang, M., Guo, Y., Li, F., Wang, X., and Fu, L. (2019). Clinical and mutation profile of pediatric patients with RASopathy-associated hypertrophic cardiomyopathy: results from a Chinese cohort. Orphanet J. Rare Dis. *14*, 29. https://doi.org/10.1186/s13023-019-1010-z.

43. Farncombe, K.M., Thain, E., Barnett-Tapia, C., Sadeghian, H., and Kim, R.H. (2022). LZTR1 molecular genetic overlap with clinical implications for Noonan syndrome and schwannomatosis. BMC Med. Genomics *15*, 160. https://doi.org/10.1186/s12920-022-01304-x.

44. Busley, A.V., Gutiérrez-Gutiérrez, Ó., Hammer, E., Steinegger, M., Böhmer, L., Schroeder, H., Kleinsorge, M., Altmüller, J., Marbach, F., Hasenfuss, G., et al. (2023). LZTR1 polymerization provokes cardiac pathology in recessive Noonan syndrome. Preprint at bioRxiv. https://doi.org/10.1101/2023.01.10.523203.

45. Jaganathan, K., Kyriazopoulou Panagiotopoulou, S., McRae, J.F., Darbandi, S.F., Knowles, D., Li, Y.I., Kosmicki, J.A., Arbelaez, J., Cui, W., Schwartz, G.B., et al. (2019). Predicting Splicing from Primary Sequence with Deep Learning. Cell *176*, 535–548.e24. https://doi.org/10.1016/j.cell.2018.12.015.

46. Cuevas-Navarro, A., Rodriguez-Muñoz, L., Grego-Bessa, J., Cheng, A., Rauen, K.A., Urisman, A., McCormick, F., Jimenez, G., and Castel, P. (2022). Cross-species analysis of LZTR1 loss-of-function mutants demonstrates dependency to RIT1 orthologs. Elife *11*, e76495. https://doi.org/10.7554/eLife.76495.

47. Kleinsorge, M., and Cyganek, L. (2020). Subtype-Directed Differentiation of Human iPSCs into Atrial and Ventricular Cardiomyocytes. STAR Protoc. *1*, 100026. https://doi.org/10.1016/j.xpro.2020.100026.

48. Greenberg, B., Butler, J., Felker, G.M., Ponikowski, P., Voors, A.A., Desai, A.S., Barnard, D., Bouchard, A., Jaski, B., Lyon, A.R., et al. (2016). Calcium upregulation by percutaneous administration of gene therapy in patients with cardiac disease (CUPID 2): a randomised, multinational, double-blind, placebo-controlled, phase 2b trial. Lancet *387*, 1178–1186. https://doi.org/10.1016/S0140-6736(16)00082-9.

49. Greenberg, B. (2017). Gene therapy for heart failure: time to go back to the drawing board. Eur. J. Heart Fail. *19*, 1542–1544. https://doi.org/10.1002/ejhf.914.

50. Mearini, G., Stimpel, D., Geertz, B., Weinberger, F., Krämer, E., Schlossarek, S., Mourot-Filiatre, J., Stoehr, A., Dutsch, A., Wijnker, P.J.M., et al. (2014). Mybpc3 gene therapy for neonatal cardiomyopathy enables long-term disease prevention in mice. Nat. Commun. *5*, 5515. https://doi.org/10.1038/ncomms6515.

51. Hu, Z., Wang, S., Zhang, C., Gao, N., Li, M., Wang, D., Wang, D., Liu, D., Liu, H., Ong, S.-G., et al. (2020). A compact Cas9 ortholog from Staphylococcus Auricularis (SauriCas9) expands the DNA targeting scope. PLoS Biol. *18*, e3000686. https://doi.org/10.1371/journal.pbio.3000686.

52. Hu, Z., Zhang, C., Wang, S., Gao, S., Wei, J., Li, M., Hou, L., Mao, H., Wei, Y., Qi, T., et al. (2021). Discovery and engineering of small SlugCas9 with broad targeting range and high specificity and activity. Nucleic Acids Res. *49*, 4008–4019. https://doi.org/10.1093/nar/gkab148.

53. Andelfinger, G., Marquis, C., Raboisson, M.-J., Théoret, Y., Waldmüller, S., Wiegand, G., Gelb, B.D., Zenker, M., Delrue, M.-A., and Hofbeck, M. (2019). Hypertrophic Cardiomyopathy in Noonan Syndrome Treated by MEK-Inhibition. J. Am. Coll. Cardiol. *73*, 2237–2239. https://doi.org/10.1016/j.jacc.2019.01.066.

54. Motta, M., Fidan, M., Bellacchio, E., Pantaleoni, F., Schneider-Heieck, K., Coppola, S., Borck, G., Salviati, L., Zenker, M., Cirstea, I.C., and Tartaglia, M. (2019). Dominant Noonan syndrome-causing LZTR1 mutations specifically affect the Kelch domain substrate-recognition surface and enhance RAS-MAPK signaling. Hum. Mol. Genet. *28*, 1007–1022. https://doi.org/10.1093/hmg/ddy412.

55. Ishikawa, K., Weber, T., and Hajjar, R.J. (2018). Human Cardiac Gene Therapy. Circ. Res. *123*, 601–613. https://doi.org/10.1161/CIRCRESAHA.118.311587.

56. Tatiossian, K.J., Clark, R.D.E., Huang, C., Thornton, M.E., Grubbs, B.H., and Cannon, P.M. (2021). Rational Selection of CRISPR-Cas9 Guide RNAs for Homology-Directed Genome Editing. Mol. Ther. *29*, 1057–1069. https://doi.org/10.1016/j.ymthe.2020.10.006.

57. Truong, L.N., Li, Y., Shi, L.Z., Hwang, P.Y.-H., He, J., Wang, H., Razavian, N., Berns, M.W., and Wu, X. (2013). Microhomology-mediated End Joining and Homologous Recombination share the initial end resection step to repair DNA double-strand breaks in mammalian cells. Proc. Natl. Acad. Sci. USA *110*, 7720–7725. https://doi.org/10.1073/pnas.1213431110.

58. Pasquini, G., Cora, V., Swiersy, A., Achberger, K., Antkowiak, L., Müller, B., Wimmer, T., Fraschka, S.A.-K., Casadei, N., Ueffing, M., et al. (2020). Using Transcriptomic Analysis to Assess Double-Strand Break Repair Activity: Towards Precise in vivo Genome Editing. Int. J. Mol. Sci. *21*, 1380. https://doi.org/10.3390/ijms21041380.

59. Rössler, U., Hennig, A.F., Stelzer, N., Bose, S., Kopp, J., Søe, K., Cyganek, L., Zifarelli, G., Ali, S., von der Hagen, M., et al. (2021). Efficient generation of osteoclasts from human induced pluripotent stem cells and functional investigations of lethal CLCN7-related osteopetrosis. J. Bone Miner. Res. *36*, 1621–1635. https://doi.org/10.1002/jbmr.4322.

60. Haeussler, M., Schönig, K., Eckert, H., Eschstruth, A., Mianné, J., Renaud, J.-B., Schneider-Maunoury, S., Shkumatava, A., Teboul, L., Kent, J., et al. (2016). Evaluation of off-target and on-target scoring algorithms and integration into the guide RNA selection tool CRISPOR. Genome Biol. *17*, 148. https://doi.org/10.1186/s13059-016-1012-2.

61. Park, J., Lim, K., Kim, J.-S., and Bae, S. (2017). Cas-analyzer: an online tool for assessing genome editing results using NGS data. Bioinformatics *33*, 286–288. https://doi.org/10.1093/bioinformatics/btw561.

62. de Sainte Agathe, J.M., Filser, M., Isidor, B., Besnard, T., Gueguen, P., Perrin, A., van Goethem, C., Verebi, C., Masingue, M., Rendu, J., et al. (2023). SpliceAI-visual: a free online tool to improve SpliceAI splicing variant interpretation. Hum. Genomics *17*, 7. https://doi.org/10.1186/s40246-023-00451-1.

OMTN, Volume *35*

## Supplemental information

# Preclinical evaluation of CRISPR-based therapies for Noonan syndrome caused by deep-intronic *LZTR1* variants

**Carolin Knauer, Henrike Haltern, Eric Schoger, Sebastian Kügler, Lennart Roos, Laura C. Zelarayán, Gerd Hasenfuss, Wolfram-Hubertus Zimmermann, Bernd Wollnik, and Lukas Cyganek**

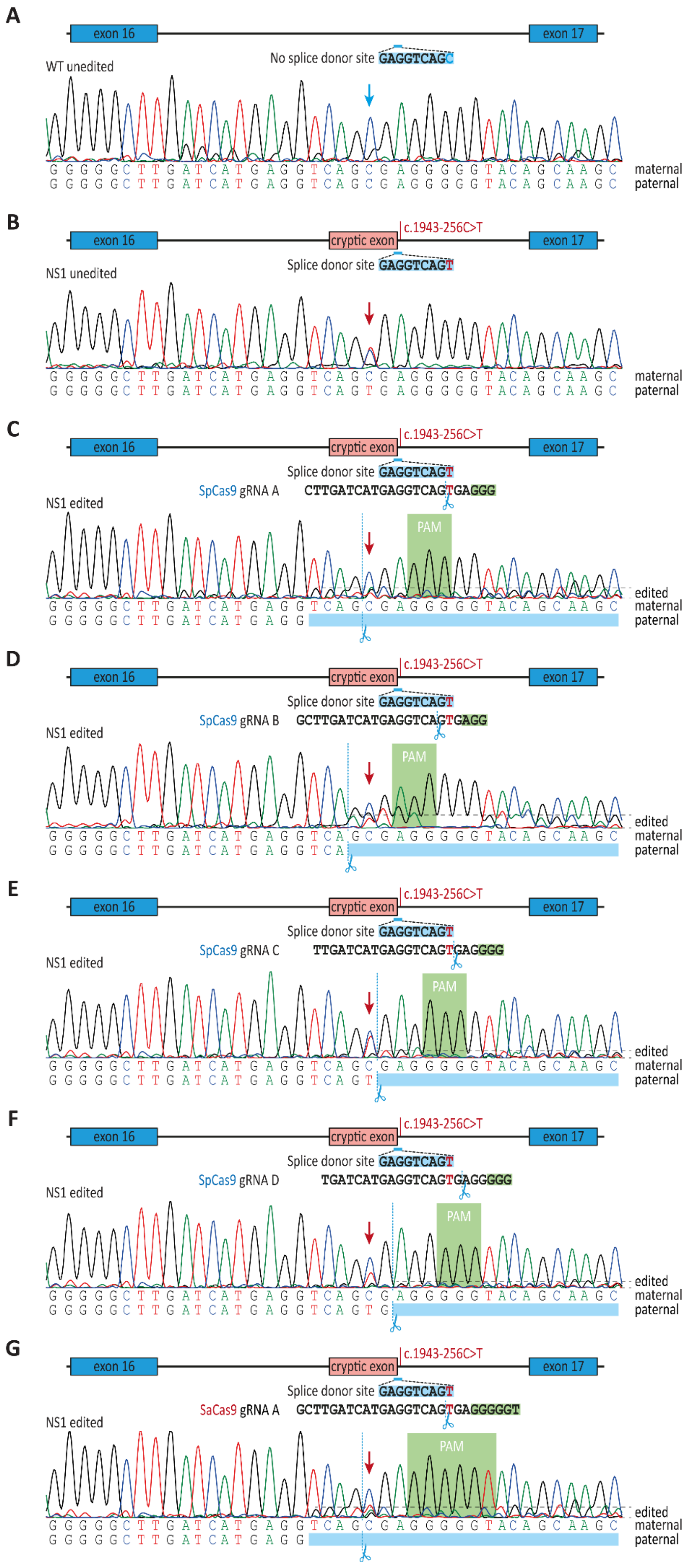
A
exon 16
exon 17
No splice donor site GAGGTCAGC
WT unedited
maternal
paternal
B
exon 16
cryptic exon
c.1943-256C>T
exon 17
Splice donor site GAGGTCAGT
NS1 unedited
maternal
paternal
C
SpCas9 gRNA A CTTGATCATGAGGTCAGTGAGGG
NS1 edited
PAM
edited
maternal
paternal
D
SpCas9 gRNA B GCTTGATCATGAGGTCAGTGAGG
NS1 edited
PAM
E
SpCas9 gRNA C TTGATCATGAGGTCAGTGAGGGG
NS1 edited
PAM
F
SpCas9 gRNA D TGATCATGAGGTCAGTGAGGGGG
NS1 edited
PAM
G
SaCas9 gRNA A GCTTGATCATGAGGTCAGTGAGGGGGT
NS1 edited
PAM

**Figure S1: Allele-specific targeting of the deep-intronic variant in *LZTR1* intron 16 by different CRISPR/Cas9 combinations in patient-specific iPSCs. (A,B)** Sanger sequencing of *LZTR1* intron 16 for WT (A) and NS1 (B). **(C-G)** Analysis of editing efficiencies of iPSCs from patient NS1 3-4 days post-transfection by Sanger sequencing for allele-specific disruption of the cryptic splice site using SpCas9 with guide RNA A (C), SpCas9 with guide RNA B (D), SpCas9 with guide RNA C (E), SpCas9 with guide RNA D (F), and SaCas9 with guide RNA A (G).

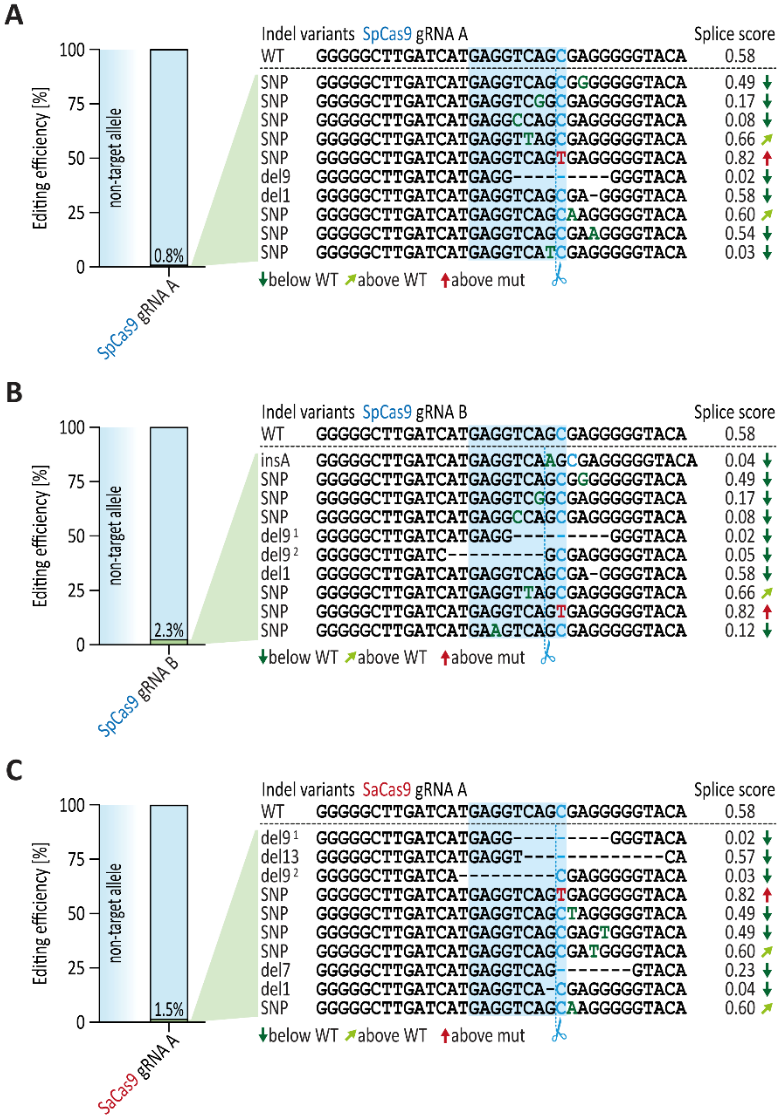


**Figure S2: Evaluation of editing specificity of the different CRISPR/Cas9 combinations targeting *LZTR1* intron 16 in WT iPSCs. (A-C)** Analysis of off-target editing of the non-mutated *LZTR1* intron 16 locus in WT iPSCs 3-4 days post-transfection using amplicon sequencing and computational prediction of splice site motifs for SpCas9 and guide RNA A (A), for SpCas9 and guide RNA B (B), and for SaCas9 and guide RNA A (C).

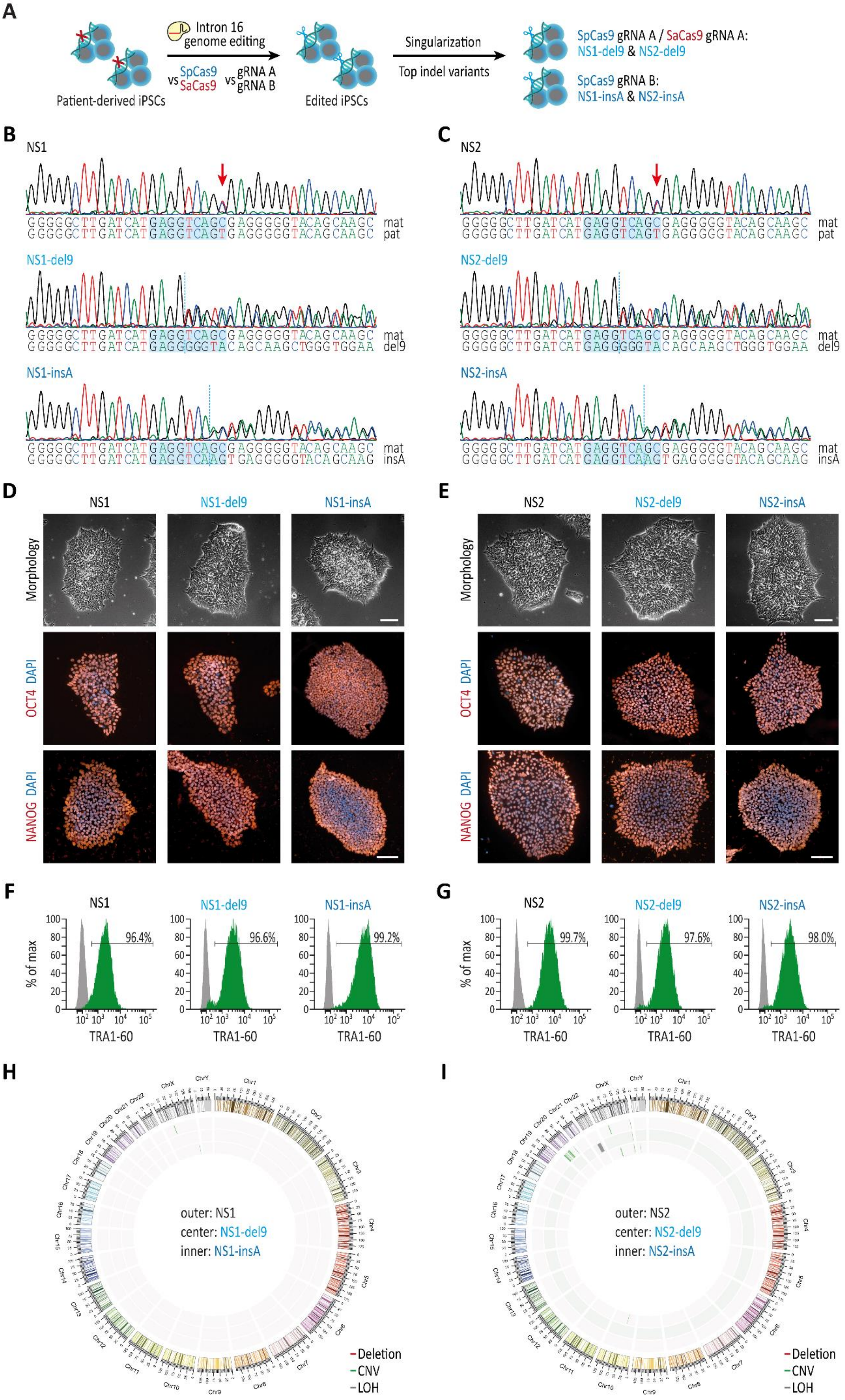
A
Intron 16 genome editing
vs SpCas9 SaCas9
vs gRNA A gRNA B
Patient-derived iPSCs
Edited iPSCs
Singularization
Top indel variants
SpCas9 gRNA A / SaCas9 gRNA A:
NS1-del9 & NS2-del9
SpCas9 gRNA B:
NS1-insA & NS2-insA
B
NS1
NS1-del9
NS1-insA
C
NS2
NS2-del9
NS2-insA
mat
pat
del9
insA
D
NS1
NS1-del9
NS1-insA
Morphology
OCT4 DAPI
NANOG DAPI
E
NS2
NS2-del9
NS2-insA
F
G
96.4%
96.6%
99.2%
99.7%
97.6%
98.0%
% of max
TRA1-60
H
I
outer: NS1
center: NS1-del9
inner: NS1-insA
outer: NS2
center: NS2-del9
inner: NS2-insA
Deletion
CNV
LOH

**Figure S3: Generation of isogenic iPSCs with top indel variants upon CRISPR/Cas9 editing of *LZTR1* intron 16 in patient-specific iPSCs. (A)** Generation of monoclonal CRISPR-edited iPSCs harboring the top indel variants for SpCas9/SaCas9 with guide RNA A and SpCas9 with guide RNA B by singularization of transfected bulks. **(B,C)** Sanger sequencing of unedited and CRISPR-edited iPSCs harboring the top indel variants 9-base pair deletion (del9) and 1-base pair insertion of adenosine (insA) derived from patients NS1 (B) and NS2 (C). **(D,E)** Patient-specific and CRISPR-edited iPSCs derived from patients NS1 (D) and NS2 (E) showed a typical human stem cell-like morphology and expressed the key pluripotency markers OCT3/4 and NANOG as assessed by light microscopy and immunocytochemistry; nuclei were counter-stained with Hoechst 33342 (blue); scale bar: 100 µm. **(F,G)** Flow cytometry analysis of pluripotency marker TRA-1-60 revealed homogeneous populations of pluripotent cells in generated iPSC lines derived from patients NS1 (F) and NS2 (G). Gray peaks represent the negative controls. **(H,I)** Molecular karyotyping using a genome-wide microarray demonstrated chromosomal stability after genome editing of iPSCs derived from patients NS1 (H) and NS2 (I). CNV: copy number variation, LOH: loss of heterozygosity.

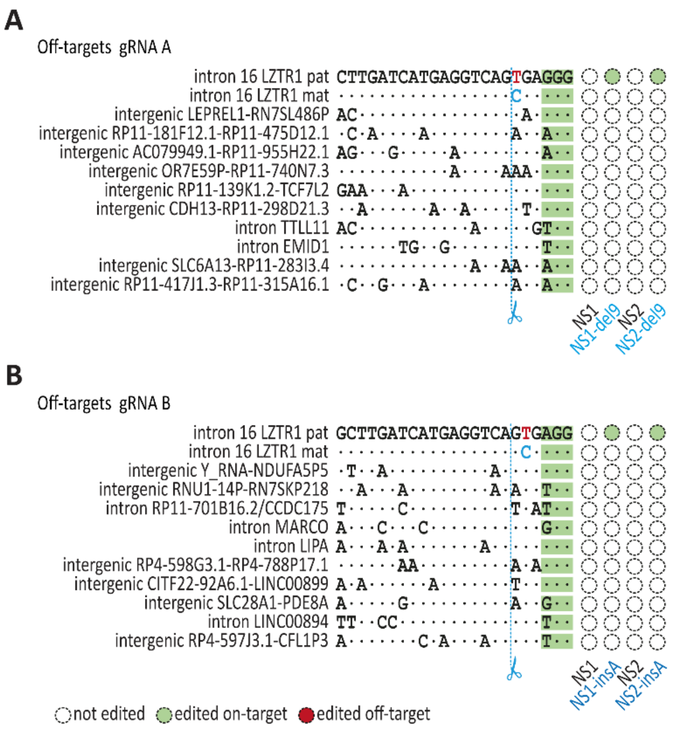


**Figure S4: Off-target screening in CRISPR-edited iPSCs. (A,B)** Sanger sequencing of the top ten predicted off-target regions, ranked by the CFD off-target score using CRISPOR, revealed no off-target editing of CRISPR/Cas9 in CRISPR-edited iPSCs compared to patient-derived cells for SpCas9 and guide RNA A (A) and SpCas9 and guide RNA B (B).

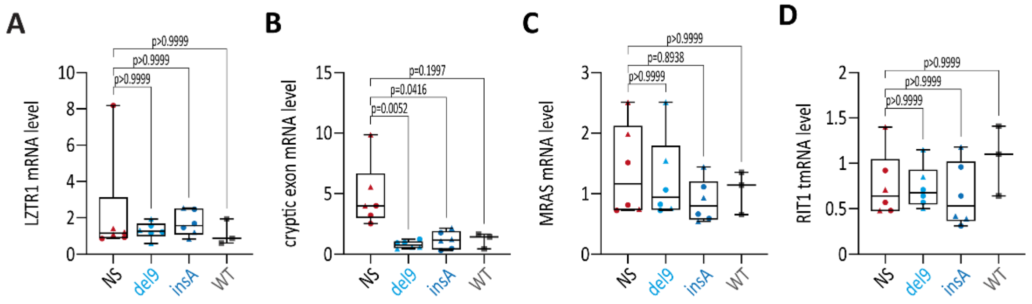


**Figure S5: Gene expression analysis in patient-specific and CRISPR-edited iPSC-CMs. (A-D)** Quantitative gene expression analysis of *LZTR1* (A), *LZTR1* transcript including the cryptic exon (B), and LZTR1 substrates *MRAS* (C), and *RIT1* (D) in patient-specific and CRISPR-edited iPSC-CMs at day 30 of differentiation, assessed by real-time PCR, revealed no expression differences at transcriptional level across all iPSC lines; samples were analyzed in triplicates and data were normalized to *GAPDH*, *RPL37A*, and *TUBB5* expression and WT controls; n=3 independent differentiations per iPSC line. Data were analyzed by nonparametric Kruskal-Wallis test with Dunn's correction and are presented as mean ± SEM (A-D).

A

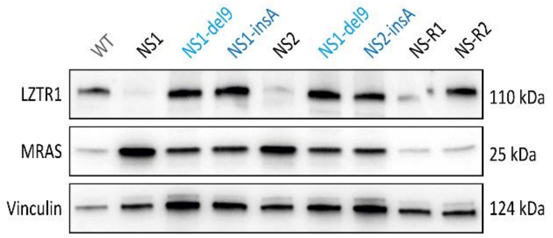


**Figure S6: Restoration of LZTR1 function and normalization of MRAS levels upon CRISPR/Cas9 editing of *LZTR1* intron 16 compared to parents' iPSC-CMs. (A)** Representative blots of LZTR1 and MRAS levels, assessed by Western blot, in patient-specific iPSC-CMs and CRISPR-corrected iPSC-CMs in comparison to iPSC-CMs from the father (NS-R1) and the mother (NS-R2) at day 30 of differentiation; Vinculin served as loading control.

A

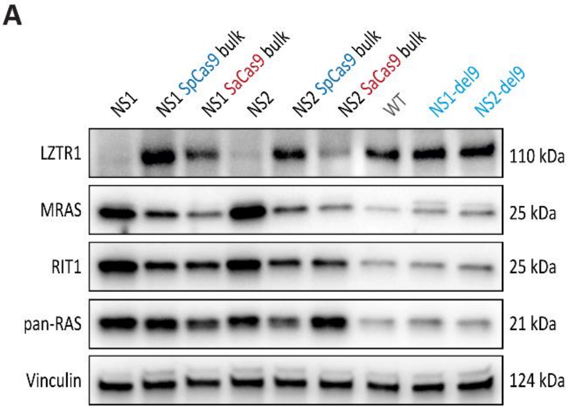


**Figure S7: Restoration of LZTR1 function and normalization of RAS GTPase levels upon CRISPR/Cas9 editing of *LZTR1* intron 16 in differentiated bulks. (A)** Representative blots of LZTR1 and RAS GTPase levels, assessed by Western blot, in iPSC-CMs at day 30 of differentiation transfected at iPSC level in comparison to unedited and monoclonal CRISPR-edited iPSC-CMs from the patients; Vinculin served as loading control.

**Table S1: Guide RNA target sequences targeting *LZTR1* intron 16.**

| **Guide RNA** | **Target sequence** |
|---|---|
| guide RNA A | (G)CTTGATCATGAGGTCAGTGA |
| guide RNA B | GCTTGATCATGAGGTCAGTG |
| guide RNA C | TTGATCATGAGGTCAGTGAG |
| guide RNA D | TGATCATGAGGTCAGTGAGG |

**Table S2: Primer sequences used for PCR, reverse transcriptase PCR and real-time PCR.**

| **Gene (gDNA)** | **Primer** |
|---|---|
| LZTR1 In16 | TGTAATGTCACAGGCCCCTT / TGTTGCTGCCCTGAACAAAA |
| Guide RNA A Off-target 1 | GCATAAAAGAAATGCCCACAA / GAAAGTAAATACAGGGAACAGAAAGC |
| Guide RNA A Off-target 2 | ATGGGTCACACACCCTCTTC / CATGTTCAGTGCTGAGTACCAA |
| Guide RNA A Off-target 3 | GCTCCAAGTTTCCTGAATAGTTT / TCTTCCGATCCAGTGTTGTG |
| Guide RNA A Off-target 4 | CTACAAGGAACCCCCAAAGG / TGAGCTCTGTGCTCTATGCAG |
| Guide RNA A Off-target 5 | TGTCACCTTTGCCCTTCTTT / CCGCCACATCTGTCTTCC |
| Guide RNA A Off-target 6 | TGAGGCTCTCAAGACACTGC / AGAAACTTGCCAACCGTGCT |
| Guide RNA A Off-target 7 | CATCCCCTGCCAGTCTAAAA / GCACAAGTCTGCAATAAATGG |
| Guide RNA A Off-target 8 | GACAACTAGGAAGCTCTTTTTGG / GGAGGAGATTCAGGACAGCA |
| Guide RNA A Off-target 9 | TCAATTGTGGCAGTGGTTTC / GGGCCCATAGCTGTTTCTTT |
| Guide RNA A Off-target 10 | TGGGGACACGTTAGTGAACA / AGGGCACCAATGATGTGTCT |
| Guide RNA B Off-target 1 | TGGGGAGAGGAATTCATGCA / CCTACAACTGCCGGTCATTG |
| Guide RNA B Off-target 2 | GCACCTCTCAATCCTCTGCT / AGCCCATCCACCAAAAGAAGA |
| Guide RNA B Off-target 3 | CGTCAAATGAGACTGAATCCCTCTA / GCCTCCCATAGTGCTAGGATTAC |
| Guide RNA B Off-target 4 | GGGCGCCACATGTTTGTATT / TGCCCCTCATCATCTCTTGC |
| Guide RNA B Off-target 5 | AAGCCTCTTCCCTGCACTAA / GCAAGGAAGGTGTTGTACTCC |
| Guide RNA B Off-target 6 | ATTTTTCAAGCCCTGTGGTAAC / AGAGACTTTTGCAATGTTCCAGT |
| Guide RNA B Off-target 7 | AGAGGCGCTGCAGTGAATTA / CAGGAACCAAGGGAAAGGCT |

| | |
|---|---|
| Guide RNA B<br>Off-target 8 | CTGCCCCAATCCATTTTGCC /<br>TCCTGGGAATGGACTGACCT |
| Guide RNA B<br>Off-target 9 | CCACAGCTGAAAAGCATCCC /<br>TGTTGGTGAGAGCCAGACAC |
| Guide RNA B<br>Off-target 10 | GTGTGGGGACAGATAAGTGGA /<br>CCTTCCTGGGAGCTTGTGTT |
| **Gene (RT-PCR)** | **Primer** |
| GAPDH | AGAGGCAGGGATGATGTTCT /<br>TCTGCTGATGCCCCCATGTT |
| LZTR1 Ex16-17 | GTAAAGGAGTCCCACTTCAAC /<br>GCAACAGAGTGATGTCACAG |
| LZTR1 Ex1 | ATGGCTGGACCGGGCAGCA /<br>ACCCACGAACTCGTCGCAGG |
| **Gene (real-time)** | **Primer** |
| GAPDH | GGAGCGAGATCCCTCCAAAAT /<br>GGCTGTTGTCATACTTCTCATGG |
| LZTR1 Ex6-7 | GCAACGCCAGGTTGAATGAC /<br>GAGCAGGTGTTCAGTTGGGA |
| LZTR1 In16-Ex17 | GGACATTGGCCCCTTCATTC /<br>TGTGCCTCATGATCAAGCCC |
| MRAS | CCACCATTGAAGACTCCTACCTG /<br>ACGGAGTAGACGATGAGGAAGC |
| RIT1 | TTCATCAGCCACCGATTCCC /<br>GCAGGCTCATCATCAATACGG |
| RPL37A | GTGGTTCCTGCATGAAGACAGTG /<br>TTCTGATGGCGGACTTTACCG |
| TUBB5 | CTGGACCGCATCTCTGTGTACT /<br>GCCAAAAGGACCTGAGCGAACA |

**Table S3: Antibodies used for Western blot, immunocytochemistry and flow cytometry.**

| **Primary antibody** | **Supplier** | **Resource ID** |
|---|---|---|
| α-actinin monoclonal mouse | Sigma-Aldrich | RRID:AB_476766 |
| β-actin monoclonal mouse | Santa Cruz | RRID:AB_1119529 |
| Cas9 polyclonal rabbit | Diagenode | RRID:AB_2715516 |
| HA-Tag monoclonal rabbit | Cell Signaling | RRID:AB_2798368 |
| LZTR1 monoclonal rabbit | Abcam | RRID:AB_3076250 |
| MLC2V polyclonal rabbit | Proteintech | RRID:AB_2147453 |
| MRAS polyclonal rabbit | Proteintech | RRID:AB_10950895 |
| NANOG monoclonal mouse | Thermo Fisher Scientific | RRID:AB_2536677 |
| OCT3/4-PE monoclonal human | Miltenyi Biotec | RRID:AB_2784442 |
| pan-RAS monoclonal mouse | Merck Millipore | RRID:AB_2121151 |
| RIT1 polyclonal rabbit | Abcam | RRID:AB_882379 |
| TRA-1-60-Alexa488 monoclonal mouse | BD Biosciences | RRID:AB_1645379 |
| Vinculin monoclonal mouse | Sigma-Aldrich | RRID:AB_477629 |
| **Secondary antibody** | **Supplier** | **Resource ID** |
| Alexa488 polyclonal goat anti-rabbit | Thermo Fisher Scientific | RRID:AB_143165 |
| Alexa555 polyclonal donkey anti-mouse | Thermo Fisher Scientific | RRID:AB_2536180 |
| HRP polyclonal donkey anti-rabbit | Sigma-Aldrich | RRID:AB_2722659 |
| HRP polyclonal donkey anti-mouse | Sigma-Aldrich | RRID:AB_772210 |

**Table S4: Plasmids used for virus production.**

| **Plasmid** | **Supplier** | **Resource ID** |
|---|---|---|
| Lenti_SaCRISPR_GFP | Addgene | RRID:Addgene_118636 |
| LentiCRISPRv2GFP | Addgene | RRID:Addgene_82416 |
| pMD2.G | Addgene | RRID:Addgene_12259 |
| psPAX2 | Addgene | RRID:Addgene_12260 |
| pAAV-CMV-SauriCas9 | Addgene | RRID:Addgene_135964 |
| pAAV-TNNT2-SauriCas9-U6-sgRNA | This study | - |
| pAAV-TNNT2-SaCas9-U6-sgRNA | This study | - |
| pAAV-TNNT2-SlugCas9-U6-sgRNA | This study | - |
